# Mechanisms of neurodynamic treatments (MONET): a protocol for a mechanistic, randomised, single-blind controlled trial in patients with carpal tunnel syndrome

**DOI:** 10.1186/s12891-024-07713-6

**Published:** 2024-07-27

**Authors:** Sierra-Silvestre E., Tachrount M., Themistocleous AC., Stewart M., Baskozos G., Schmid AB.

**Affiliations:** 1 Nuffield Department of Clinical Neurosciences, John Radcliffe Hospital, West Wing Level 6 OX39DU, 01865 223254 Oxford, UK; 2 Wellcome Centre for Integrative Neuroimaging, FMRIB, Nuffield Department of Clinical Neurosciences, University of Oxford, Oxford, UK

**Keywords:** Mechanisms, Neurodynamic, Carpal tunnel syndrome, Exercises, MRI, Quantitative sensory testing

## Abstract

**Background:**

Physiotherapeutic management is the first-line intervention for patients with entrapment neuropathies such as carpal tunnel syndrome (CTS). As part of physiotherapy, neurodynamic interventions are often used to treat people with peripheral nerve involvement, but their mechanisms of action are yet to be fully understood. The MONET (mechanisms of neurodynamic treatment) study aims to investigate the mechanisms of action of neurodynamic exercise intervention on nerve structure, and function.

**Methods:**

This mechanistic, randomised, single-blind, controlled trial will include 78 people with electrodiagnostically confirmed mild or moderate CTS and 30 healthy participants (*N* = 108). Patients will be randomly assigned into (1) a 6-week progressive home-based neurodynamic exercise intervention (*n* = 26), (2) a steroid injection (= 26), or (3) advice (*n* = 26) group. The primary outcome measure is fractional anisotropy of the median nerve at the wrist using advanced magnetic resonance neuroimaging. Secondary outcome measures include neuroimaging markers at the wrist, quantitative sensory testing, electrodiagnostics, and patient reported outcome measures. Exploratory outcomes include neuroimaging markers at the cervical spine, inflammatory and axonal integrity markers in serial blood samples and biopsies of median nerve innervated skin. We will evaluate outcome measures at baseline and at the end of the 6-week intervention period. We will repeat questionnaires at 6-months. Two-way repeated measures ANCOVAs, followed by posthoc testing will be performed to identify differences in outcome measures among groups and over time.

**Discussion:**

This study will advance our understanding of the mechanisms of action underpinning neurodynamic exercises, which will ultimately help clinicians to better target these treatments to those patients who may benefit from them. The inclusion of a positive control group (steroid injection) and a negative control group (advice) will strengthen the interpretation of our results.

**Trial registration:**

NCT05859412, 20/4/2023.

**Supplementary Information:**

The online version contains supplementary material available at 10.1186/s12891-024-07713-6.

## Background

Clinical guidelines recommend a conservative approach to entrapment neuropathies in people who present with mild to moderate symptoms [1–3]. Regardless of the type of entrapment neuropathy, the leading conservative treatments include steroid injection, and physical therapy [4, 5]. Various physical therapy modalities are offered as first-line interventions to manage symptoms before more invasive options (e.g., surgery) are considered [6–8]. Neurodynamic interventions have been incorporated as part of the physical therapy management of patients with peripheral nerve injuries, including entrapment neuropathies [8–10].

Neurodynamic interventions are aimed at restoring the homeostasis in and around the nervous system, by gliding the nerve in relation to its surrounding tissue while minimising neural strain [11–13]. These neurodynamic interventions facilitate the movement between neural structures and their surroundings (interface) by using a combination of joint movements or exercise [12]. Their efficacy has been confirmed as part of a combined intervention with education and splinting in some people with carpal tunnel syndrome (CTS) [14]. The uncertainty about the magnitude and specificity of these improvements in the wider population with CTS could be addressed by better understanding the mechanisms of action of neurodynamic exercises to identify those most likely to benefit.

Preclinical studies in animal models have investigated the mechanism of action of neurodynamic treatments suggesting an anti-inflammatory effect by reducing proinflammatory cytokines [15] and glial activation [16, 17]. There is also increasing in-vivo and in-vitro evidence that they may have a pro-regenerative and/or anti-degenerative effect on peripheral nerves [18–20] and can reduce the intraneural scar formation in a model of chronic constriction injury [21]. In humans, neurodynamic exercises in combination with other conservative treatments modified mechanical properties of peripheral nerves (i.e., decreased nerve stiffness) [22] and improved sensory and motor conduction velocities [23] along with pinch and grip strength [24]. Our previous study [25] showed changes in intraneural oedema using neurodynamic exercises only, but apart from T2 signal intensity, no advanced imaging or other mechanistic markers were included. How neurodynamic exercises in isolation can contribute to the pro-regenerative effect on peripheral nerves, changes in somatosensory function and the dispersion of inflammatory by-products are yet to be explored [25, 26].

A better understanding of how neurodynamic interventions work is an important first step towards personalised and precision physical therapy. We have previously shown that a set of eight nerve and tendon gliding exercises have a positive impact on symptoms [14] and can reduce MRI signals of oedema within the affected median nerve in patients with CTS [25]. CTS is an ideal model system to explore the mechanisms of action of neurodynamic treatments. It is the most common entrapment neuropathy [27] and provides good access to investigations of the function and structure of the affected nerve. We have demonstrated the presence of nerve degeneration and regeneration [26], intraneural [25], and systemic inflammation [28] using CTS as a model system. Importantly, patients with CTS are routinely treated with steroid injection (a potent anti-inflammatory medication), which has established short term benefits [29] and can therefore serve as a positive control treatment.

This single-blind randomised mechanistic controlled trial therefore uses CTS as a model system to investigate the mechanisms of action of a 6-week progressive home-based neurodynamic exercise intervention on nerve function, structure and neuroinflammation in patients with CTS compared to a positive control intervention (steroid injection) and a negative control intervention (advice). We will also include a healthy control group who will provide normative data on nerve structure, function, and inflammatory markers. The deep and comprehensive phenotyping of our patient cohort includes different advanced neuroimaging sequences (i.e., diffusion, T2 mapping and anatomical images with magnetisation transfer preparation) that combined with our functional (i.e., quantitative sensory testing, nerve conduction studies, serum protein levels in blood) could help disentangle the mechanisms of action of neurodynamic exercises locally. In future analyses, we will explore whether these neurodynamic exercises have an effect proximally at the level of the dorsal root ganglia (DRG).

## Methods

This randomised, mechanistic, single blind, controlled trial will be carried out at Oxford University, UK, and will be reported according to the CONSORT guidelines [30].

### Participants

We will include people with a confirmed diagnosis of mild or moderate of CTS based on clinical [31] and electrodiagnostic [32] criteria.

We will identify eligible patients from the neurophysiology department at Oxford University Hospitals Foundation NHS Trust (OUH), primary care services and through public advertisement (flyers, leaflets, community notice boards, emailing lists and social media). An age and gender matched cohort of healthy volunteers recruited through flyers, leaflets, community notice boards, emailing lists and social media will be included to establish normative data.

Inclusion criteria for people with CTS and healthy controls include being 18 years or older, willing, and able to give informed consent for participation in the study, and having sufficient command of the English language to complete questionnaires and the detailed assessments. The electrodiagnostic testing will include median, ulnar and radial nerve sensory and motor studies to determine the presence of CTS and the absence of other peripheral neuropathies, according to established protocols [32]. The temperature of the hand will be standardised before testing to > 31 °C. Sensory nerve action potential latencies, amplitudes and nerve conduction velocities will be recorded orthodromically over the wrist for the median (index finger), ulnar (little finger) and superficial radial nerve (snuffbox). Compound muscle action potentials will be registered for the median nerve (abductor pollicis brevis stimulated from the wrist and antecubital fossa), and ulnar nerve (adductor digiti minimi stimulated from the wrist, below and above the elbow). Additionally, we will record orthodromic sensory nerve action potentials by stimulating the ring finger and register the response at the wrist. The presence of a ‘double peak’ (increased latency of median sensory nerve action potential compared to ulnar sensory nerve action potential) will be classified as abnormal [33]. The motor latency difference between the median nerve (second lumbrical) and ulnar nerve (palmar interossei) will be assessed over a fixed distance of 8 cm with a delay of the median motor potential relative to the ulnar latency > 0.4 ms deemed abnormal [34].

The severity of CTS will be graded according to Bland’s criteria [32]. Only patients with mild (sensory conduction velocity from index finger to wrist < 40 m/s with motor terminal latency from wrist to abductor pollicis brevis [APB] < 4.5 ms) and moderate CTS (motor terminal latency > 4.5ms and < 6.5ms with preserved index finger sensory nerve action potential) will be included. People with severe CTS are recommended surgery according to clinical guidelines [1, 2] and thus will not be included in this study.

People with CTS will be excluded if they had previous ipsilateral CTS surgery (patients with unilateral surgery on the non-study hand are eligible to participate) or are planning to undergo surgery in the next 6 weeks, had a steroid injection for their CTS in the 6 months prior to the study enrolment or who had already more than 1 steroid injection, have an electrodiagnostic test that reveals abnormalities other than CTS (e.g., ulnar neuropathy), present with another medical condition affecting the upper limb or neck (e.g., rheumatoid arthritis, cervical radiculopathy or myelopathy), a history of significant trauma to the upper limb or neck, diabetes, hypothyroidism, severe anxiety or depression, altered coagulation (e.g., haemophilia) or having strong anticoagulant medication that prevents skin biopsies, contraindications for steroid injections (e.g., infection of the skin, allergy to any components of the injection) or for MRI (e.g., metallic implants), or those who are pregnant, lactating or planning pregnancy during the course of the study. Healthy participants will be excluded if they present with a history of hand, arm or neck pain in the past three months, abnormalities in nerve conduction studies suggestive of CTS, or with a systemic medical condition.

### Study procedure

Consented participants (see ethics below) will receive the baseline set of questionnaires to complete before the first baseline appointment at the Nuffield Department of Clinical Neurosciences, University of Oxford. This first appointment will include a detailed bedside neurological assessment and measures of nerve function. If participants present with bilateral symptoms of CTS, the most affected hand will be evaluated. At this point, participants not meeting the inclusion criteria will be excluded from the study (e.g., severe electrodiagnostic test findings). Eligible participants will provide a blood sample and a finger skin biopsy.

**Fig. 1:**
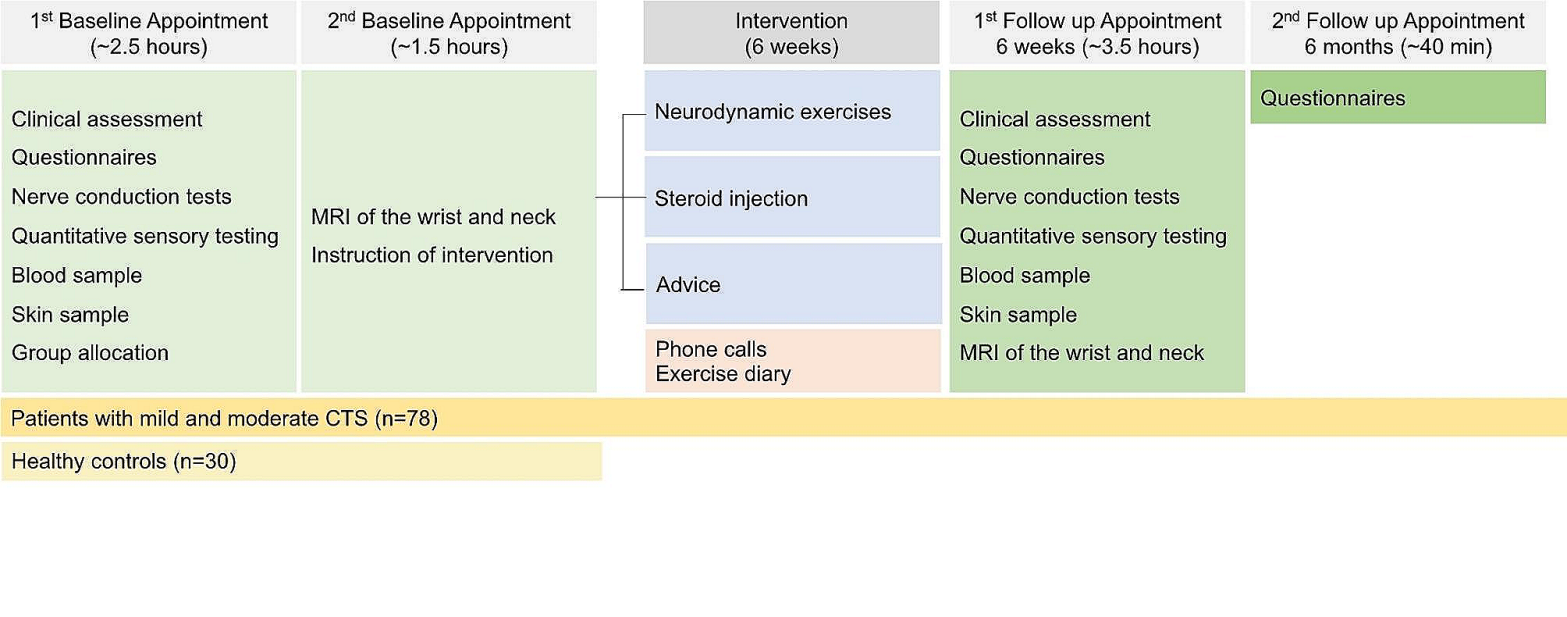
Trial flow chart

Participants will be invited for a second baseline appointment for an MRI of the wrist and the neck. Following the scan, patients will receive their assigned intervention. After the 6-week intervention period, patients will be invited for a follow-up appointment to repeat the same assessments. Finally, questionnaires will be sent out to patients at 6 months (Fig. 1).

Healthy controls will only attend the baseline appointments during which the same measures will be performed as in patients.

### Assignment of interventions and blinding

Patients will be randomly assigned to one of three interventions after their first baseline appointment. Assignment will be randomised and stratified by electrodiagnostic test severity (mild/moderate) using an online tool (https://www.randomizer.org) and implemented in REDcap. The allocation ratio will be 1:1:1. The examiner performing the outcome measures will be blinded. The central research team, including the statistician, will also be blinded.

### Interventions

#### Neurodynamic exercises

**Fig. 2:**
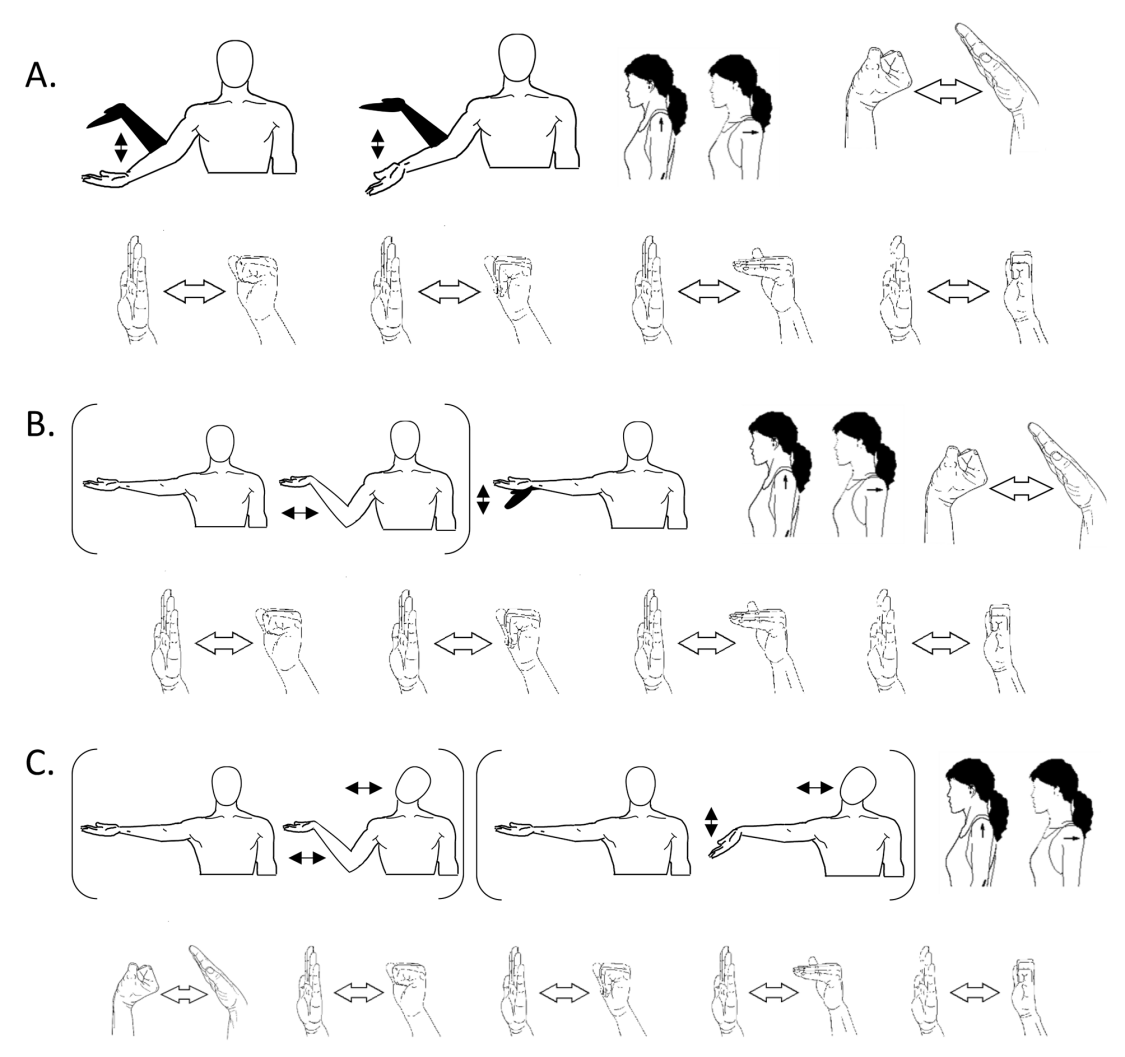
Progressive home-based programme of nerve and tendon gliding exercises (**A**) Weeks 1–2, (**B**) weeks 3–4, and (**C**) weeks 5–6. The brackets surrounding two figures indicate the combination of movements for that exercise, from the start to the end point

The ‘active’ intervention is a progressive neurodynamic mobilisation adapted from our previously established protocol [35, 36]. Patients will attend a single session (~ 30 min) with an investigator who will instruct them the home exercise programme consisting of eight nerve and tendon gliding exercises (Fig. 2). The investigator will train patients with the help of a set of videos demonstrating the exercises on either the right or the left hand, as suggested by our patient partners. Patients will be asked to perform 10 repetitions of each exercise six times a day for six weeks (~ 1.5 min per session) in a manner that does not increase symptoms. These exercises are progressed on week 3 and 5th to increase the nerve gliding or tension as per each patient’s tolerance (Fig. 2, B and C, exercises in brackets). Patients will receive a leaflet detailing the neurodynamic exercises during their second baseline appointment (Suppl. Information. Appendix A), an exercise diary as well as a link to the exercise videos in weeks 1, 3 and 5. They will also be instructed to keep performing their usual activities but not start any new treatments during the 6 weeks of the study intervention (Suppl. Information. Appendix A).

#### Steroid injections

Steroid injections are routine first-line treatment for patients with CTS [37] and will be used as a positive control group as they have established short term benefits [38, 39] and change nerve structure [40]. Patients in this group will receive a single injection of 40 mg depomedrone suspended in polyethylene glycol into the carpal tunnel (extraneurally) using the landmark technique [41]. Briefly, the needle will be inserted into the proximal carpal tunnel at the distal wrist crease immediately ulnar to the palmaris longus tendon [41]. A trained medical doctor will perform this technique as per standard practice at OUH NHS Trust. Participants will receive an information leaflet, and will be instructed to keep performing their usual activities but not start any new treatments during the 6 weeks of the study intervention (Suppl. Information Appendix B).

#### Advice group

The advice group will serve as a negative control group and will receive no additional intervention during the 6-week intervention period. Patients randomly assigned to this group will meet the investigator, who will provide them with an information leaflet detailing the advice (Suppl. Information Appendix C). Patients will be instructed to keep performing their usual activities but not start any new treatments during the 6 weeks of the study intervention.

Patients allocated to the advice group will be offered the 6-week home based exercise program as post-trial care (end of the 6-week treatment period) if they wish.

### Primary mechanistic outcome measure

#### Magnetic resonance neurography (MRN): Fractional Anisotropy (FA)

All participants will attend a MRN session on a 3 Tesla MAGNETOM Prisma scanner (Siemens, Germany) to visualise the median nerve at the wrist using a dedicated 16-channel wrist coil (Siemens, Germany).

Participants will be positioned in a ‘superman’ position, lying prone with the wrist above their head resting in the centre of the bore. The protocol includes the acquisition of multishell (b = 0, b = 300 and 800 s/mm^2^) diffusion-weighted imaging scans. In addition, true fast imaging with steady-state free precession (TRUFI) with/without magnetisation transfer (MT) preparation, and T2-mapping scanning will be acquired (see other markers in MRN). Scan parameters are summarised in Table 1.

**Table 1:** MRI sequence parameters at the wrist

	3D TRUFI	T2 mapping	Diffusion Weighted Imaging (DWI)
Repetition time (TR)	10.1 ms	7,110 ms	4,000 ms
Echo time (TE)	4.14 ms	14.7–147 ms	47 ms
Flip angle (FA)	20°	Variable	90° and 180°
Field of view (FoV)	74.3 mm	225 mm	160 mm
Dimensionality	3D	2D	2D
Slice thickness	0.6 mm	1 mm	1 mm
Number of slices	176	50	50
In-plane resolution	0.1 × 0.1 mm	0.5 × 0.5 mm	1 × 1 mm
Echo spacing	N/A	N/A	0.96ms
Fat suppression	No fat saturation	Fat saturation	Fat saturation
Parallel acquisition technique	GRAPPA factor 2	GRAPPA factor 2	GRAPPA factor 2
Bandwidth	350 Hz/Px	132 Hz/Px	1220 Hz/Px
b values	N/A	N/A	0 (NSA = 8), 300 (NSA = 5), 800 (NSA = 8)
No. signal averages	1	1	8 (b = 0), 5 (b = 300), 8(b = 800)
Scan time single phase-encode blip (right-left or left-right)	N/A	N/A	6.42, 7.42
Total scan time (min)	10.16	10.32	14.18

N/A: not applicable; NSA: number of signal averages

All diffusion image pre-processing will be performed using the FMRIB Software Library (FSL) Diffusion Toolbox v 6.0 (Oxford, UK). Briefly, TOPUP [42, 43] and EDDY [44] tools will be applied to correct for distortions and eddy currents followed by DTIFIT [45] to compute the diffusion tensor model. FLIRT will be used to co-register the diffusion metrics to the anatomical sequences.

Median nerve region of interests will be determined at three levels: the distal radio-ulnar joint (proximal), pisiform (mid-carpal tunnel) and hook of hamate (distal carpal tunnel). Fractional anisotropy (no units) will be computed as our primary outcome measure.

### Secondary mechanistic outcome measures

#### Other markers in MRN of the median nerve at the wrist

The MRN protocol of the wrist includes the acquisition of true fast imaging with steady-state free precession (TRUFI) with/without MT preparation and a multi-spin-echo scan for T2-mapping scanning (Table 1). Multi-spin-echo and TRUFI will be processed using a custom-made Python script. TRUFI volumes acquired with different phases will be combined by calculating the square root of the sum of the squares of each volume. T2 maps will be obtained from the multi-spin-echo volumes using non-linear least-squares regression. FLIRT will be used to co-register the T2 maps to the high resolution anatomical images.

MR outcome measures of the median nerve will be computed at the three levels of the carpal tunnel described for the primary outcome measure (i.e., proximal, mid, and distal carpal tunnel). In structural images, we will compute the cross-sectional area (mm^2^) and flattening ratio (arbitrary units). Mean diffusivity (mm^2^/s), and radial/axial diffusivity (mm^2^/s) will be obtained from diffusion images. Finally, T2 (ms), and magnetisation transfer ratio (arbitrary units) will be computed from their respective sequences. Metrics derived from multishell data will be explored.

#### Somatosensory function

We will use the standardised Quantitative Sensory Testing (QST) battery of the German Network for Neuropathic pain [46] to evaluate the somatosensory function over the median nerve territory of the studied hand.

Thermal thresholds will be explored with series of three repetitions to compute the average temperature (°C) for cold and warm detection thresholds (CDT, WST), hot and cold pain threshold (HPT, CPT) and five repetitions for thermal sensory limen (TSL) using a Thermotester (Somedic, Sweden, 25 × 50 mm thermode). Paradoxical heat sensations (PHS) will be recorded during TSL testing. Pain ratings during HPT and CPT will be recorded on an 11-point numerical rating scale (NRS), from ‘0’ representing no pain to ‘10’, worst pain imaginable and averages used for analyses.

Mechanical detection thresholds (MDT) will be assessed using five series of ascending/descending von Frey monofilaments (mN, geometric mean calculated). Mechanical pain threshold (MPT) will be explored with a set of weighted pinpricks (mN, geometric mean calculated). Mechanical pain sensitivity will be examined with a NRS of 0–100 using a shortened protocol of two sets of seven pseudo-random pinprick stimulations [47]. Dynamic mechanical allodynia (DMA) will be explored [26, 48] using a cotton wisp, cotton wool tip, and a standardised brush (Somedic, Sweden) [46] that will be interleaved during the MPS assessment. The geometric mean will be calculated for MPS and DMA. Wind-up ratio will be calculated as the average pain rating on a 100-NRS scale between three trains of 10 pinprick stimuli divided by three single stimuli. Vibration detection threshold (VDT) will be determined as a disappearance threshold through the average of three repetitions using a Rydel Seiffer tuning fork (64 Hz, 8/8 scale). Pressure pain threshold will be determined as the average of three series of ascending stimulus intensities (kPa) using a manual algometer (Wagner Instruments, USA).

All stimuli will be first demonstrated over the lateral, proximal forearm (radial territory) of the non-studied side, with the exception of VDT which be shown over the ulnar styloid. All thermal and mechanical stimuli will then be assessed over the palmar aspect of the index finger (proximal phalanx), except VDT (over the second metacarpophalangeal head) and PPT (over the thenar eminence). Additionally, pain thresholds including HPT, CPT, MPT and PPT will be assessed in the contralateral lower limb (upper anterolateral aspect of the tibialis anterior muscle) to determine potential effects on generalised hypersensitivity.

QST outcome measures will be transformed into Z-scores [46] using our healthy control participants as well as existing control data sets [26, 49], who will be matched for age and sex. Additionally, we will classify each participant with CTS to a specific and unique somatosensory profile [50]: (1) sensory loss; (2) thermal hyperalgesia; or (3) mechanical hyperalgesia.

#### Neurophysiological function

We will use surface electrodes to measure the median SNAP amplitudes (µV), CMAP amplitudes (mV) and conduction velocities (m/s), as described above [32].

#### Neurodynamic tests

We will explore the mechanosensitivity of the median, ulnar and radial nerve using Upper Limb Neurodynamic Tests (ULNT) in patients bilaterally [12]. The median nerve bias test (ULNT 1) will be performed with the patient lying supine and the movement sequence will involve maximum end range shoulder abduction, wrist and finger extension, forearm supination, shoulder external rotation and elbow extension [12]. The ulnar nerve bias (ULNT 3) sequence starts with the patient lying supine and their arm resting by the side followed by wrist extension, forearm pronation, elbow flexion, shoulder external rotation, shoulder girdle depression and shoulder abduction [12]. The radial nerve bias (ULNT 2B) starts with the patient in supine and diagonally on the plinth. The sequence involves shoulder girdle depression, elbow extension, forearm pronation, wrist and finger flexion, followed by shoulder abduction [12]. The end point of these tests is the first onset of symptoms (P1). The test will be considered positive if (1) patient’s symptoms are at least partially reproduced, and (2) structural differentiation (away from the site of pain) changes the symptoms [51].

We will also quantify the elbow extension angle at P1 with an inclinometer in a slightly modified ULNT1 manoeuvre. The testing will involve shoulder at 90 degrees abduction, neutral shoulder rotation, wrist and fingers extension and finally, elbow extension. This assessment will be performed in patients and healthy participants (reference values).

#### Pinch grip strength

Tip to tip pinch of the thumb and index finger, key pinch between the radial side of index finger and thumb, and tripod pinch using the thumb, index and middle finger (kg, averaged 3 repetitions) will be assessed using a mechanical pinch gauge dynamometer bilaterally (B&L Engineering, CA, USA) as per standard recommendations [52, 53].

#### Self-reported outcome measures

We will use a battery of validated questionnaires to evaluate patients’ symptoms, functional deficits, quality of life, sleep, and psychological co-morbidities at all time points. Patients assigned to the neurodynamic exercise group will be asked to complete an exercise diary daily (electronically or on paper) to monitor adherence for the duration of the intervention (6-weeks). Healthy participants will only complete questionnaires determining function, quality of life, sleep and psychological comorbidities (Table 2).

**Table 2:** List of questionnaires

Questionnaire	Type of outcome measure	Baseline	6 weeks	6 months
*Assessment of pain intensity and location*: • Boston Carpal Tunnel questionnaire [59] • Paper based or electronic body diagram to localise symptoms • Subject reported average intensity of pain, paraesthesia and numbness. Reported on 10 cm visual analogue scales ranging from no symptoms to worst symptoms ever. • Central sensitisation index (CSI) [60]	Secondary Descriptive Descriptive Secondary	x	x	x
*Assessment of functional deficits*: • Quick DASH [61] (healthy) • Patient specific functional scale [62, 63]	Secondary	x	x	X
*Neuropathy Screening tools*: • DN4 [64] • painDETECT [65]	Secondary	x	x	x
*Assessment of neuropathic pain symptoms*: • Neuropathic Pain Symptom Inventory [64]	Secondary	x	x	x
*Assessment of psychological co-morbidity*: • Depression Anxiety Positive Outlook Scale (DAPOS) [66] (healthy) • Pain Catastrophising Scale (PCS) [67] (healthy) • Short-form Pain Anxiety Symptoms Scale (PASS-20) [68] (healthy)[62]	Secondary	x	x	x
*Assessment of sleep interference*: • Insomnia Severity Index [69] (healthy)[62]	Secondary	x	x	x
*Assessment of quality of life*: • EQ-5D-5 L [70] (healthy)	Secondary	x	x	x
*Assessment of longitudinal change*: • Global rating of change questionnaire [71]	Secondary		x	x
*Assessment of medication/diagnoses*: • Medications/diagnoses questions (see unvalidated questionnaires) (healthy)	Descriptive	x	x	x

All listed questionnaires are to be completed by patients. After the name of the questionnaire, we have indicated in brackets which ones will also completed by healthy control participants

### Exploratory outcomes

#### Blood inflammatory markers (e.g., cytokine panels)

We will collect 26 ml of venous blood from antecubital venepuncture. Serum will be extracted from whole blood collected into BD Vacutainer SST tubes (gold cap) and centrifuged at 1.3 g for 10 min at 4 °C 30 min after venepuncture. Serum fraction will be immediately frozen at -80 degrees and stored for batch processing in accordance with the Human Tissue Act. In future analyses, we can explore the concentrations of serum inflammatory protein levels and other markers of interest, including but not limited to TGF-β, CCL5, and IL-4, as per our previous work [28].

For future analyses and biobanking, we will also sample blood into RNA stabilising tubes (Tempus™ blood RNA tube, Fisher Scientific), serum clot activator tubes (red cap BD, Wokingham UK) and EDTA containing tubes (lavender cap, BD, Wokingham UK).

#### Cutaneous markers (e.g., inflammatory markers, innervation markers)

Future exploratory analysis will involve looking at the presence of inflammatory and innervation markers via 3 mm skin punch biopsies performed on the ventrolateral side of the proximal phalanx of the index finger [48] (baseline and 6 weeks in patients, baseline only in healthy controls). In patients, the 6-weeks biopsy will be performed slightly more proximal, avoiding the primary biopsy site as we have previously done [26]. Samples will be processed for immunostaining (fixation in fresh periodate-lysine-paraformaldehyde for 30 min before being washed in 0.1 M phosphate buffer and cryoprotected in 15% sucrose in 0.1 M phosphate buffer and freezing in optimal cutting temperature gel at -80 degrees) and molecular experiments (snap freezing in liquid nitrogen before storing at -80 degrees).

#### MRN markers at the DRG

As preclinical literature suggests changes at the level of the DRG after peripheral nerve injury [54, 55], all participants will be scanned to visualise their cervical DRG with a 64-channel head/neck coil (Siemens, Germany). Participants will be lying supine. The protocol includes T2 weighted (T2W) and Diffusion Weighted (DW) scans. T2W and DW images will be acquired using Sampling Perfection with Application optimised Contrast using different flip angle Evolution (SPACE) and Readout Segmentation Of Long Variable Echo trains (RESOLVE) sequences, respectively. DW images will be registered to T2W images (Supplementary Table 1).

MR outcome measures will be obtained from structural images, such as volume (mm^3^) for the DRG. FA, mean diffusivity (mm^2^/s), and radial/axial diffusivity (mm^2^/s) will be calculated from diffusion images. Metrics derived from multishell data will be explored.

### Additional cohort characterisation

#### Demographic variables

We will collect demographic data on age, sex, height, weight, ethnicity, profession, working status, and years of education. Medical information will include the most affected side, presence of unilateral/bilateral symptoms, duration of symptoms, previous history of carpal tunnel syndrome (personal and in the family), previous treatment received for carpal tunnel syndrome (CTS), current and previous medications, smoking and alcohol intake.

#### Clinical assessment

Patients will complete two body charts, one reflecting the presence of symptoms in their body, and a detailed diagram of hand symptoms. Patients will undergo a detailed neurological examination which will include upper limb myotome testing (C4-T1) recorded on the five-point British Medical Research Council scale (M0 = no contraction, M1 = flicker or trace of contraction; M2 = active movement, with gravity eliminated; M3 = active movement against gravity; M4 = active movement against gravity and resistance; M5 = normal power) [56]; biceps, triceps and brachioradialis reflexes recorded as normal, absent, reinforced or hyperreflexia (adapted from National Institute of Neurological Disorders and Stroke Scale (NINDS) [57], and exploring potential loss of sensation to light touch and pinprick recorded as absent, reduced, normal or increased on two separate body charts.

### Safety and adherence to neurodynamic exercises

Patients assigned to the neurodynamic exercise group will be asked to complete an electronic (or paper-based) exercise diary daily to monitor treatment adherence for the duration of the intervention (6-weeks). Patients will be asked to indicate how many times they perform the exercises each day.

We will also use periodic phone calls during the 6-week study period (3rd day after randomisation, weekly thereafter) to check on progress in each of the intervention groups, decrease study attrition, give advice on exercise performance when needed, check if there are potential adverse events associated with the exercise intervention or the steroid injection, and to confirm that they have not started any new treatments during the intervention period in the steroid and advice groups. This was suggested by our patients’ partners to increase adherence.

### Sample size calculation

Sample size is based on our previous MRI study demonstrating intraneural signal reduction after neurodynamic exercises [25]. Conservatively assuming a 30% smaller effect size (within subjects repeated measures ANOVA with 3 groups & 2 time points), *n* = 63 patients are required (*n* = 21 per group, d = 0.22, power = 80%, alpha = 0.05). To account for a 20% drop out rate, we will include 78 participants (26 per group). We will include 30 healthy participants to establish normative data.

### Statistical analysis

Data will be analysed using R Studio (v 7.2, RStudio, Boston, USA) [58]. The distribution of the data will be checked for normality, and parametric, or non-parametric methods will be used as appropriate. Participants’ characteristics in each group will be described at baseline.

Two-way repeated measures ANCOVAs (factors time and intervention, adjusted for baseline measurements as a continuous covariate) followed by post-hoc testing will be performed to identify differences in primary/secondary outcome measures among groups and over time. If there is deviation from normality, we will use non-parametric alternatives (i.e. ranked ANCOVA).

If the missing values mechanism is likely to be missing at random (MAR) or missing completely at random (MCAR), we will perform multiple imputation by chained equations. The appropriate model will be fitted in all the completed datasets, then coefficient estimates and standard errors will be pooled across imputations using Rubin’s rules.

The level of statistical significance will be set at *p* = 0.05. Adjustments for multiple comparisons will be used as appropriate.

### Data management

All study data will be entered on paper or directly onto EXCEL or REDCap database. The name and any other identifying details will not be included in any study data electronic file, with the exception of REDCap to send out reminders to complete follow up questionnaires.

Data entries will be randomly checked for accuracy by an independent researcher (up to 30%). All data will be kept on firewall and password-protected computers and any paper information will be stored safely in lockable cabinets in a swipe-card secured building and would only be accessed by the research team. MRI data will be stored in a secure University server.

As this a mechanistic study and not a clinical trial, we do not have an external monitoring committee but we will internally monitor and regularly audit data collection and delivery of interventions. No interim analysis will be done of the longitudinal data. All research data and records will be stored in accordance with data protection and University policies.

### Ethics and dissemination

Protocol amendments will be approved by the ethics committee and then disseminated to site investigators via meetings and updating of study resources and guidelines. Changes in the protocol will be reported to the trial registry and mentioned in future publications.

### Patient and public involvement (PPI)

Patient partners highlighted the relevance of this study. They were involved in the design of the videos in the neurodynamic exercise group (i.e., including demonstrative videos with either right or left hand) and how these videos should be displayed (i.e., individual videos explaining each exercise and a summary video with the full sequence). Additionally, they provided feedback on the potential burden of the duration of the baseline assessment and suggested two separate appointments (i.e., MRI on a separate day). They agreed on the feasibility to perform the sessions suggested in the home-based neurodynamic intervention. They suggested ways to optimise the adherence to the neurodynamic intervention by facilitating access to the home-based program using different devices (i.e., computer or mobile phones), and the use of electronic and paper-based exercise diaries along with daily email reminders to input the number of exercise sessions per day.

Patient partners will continue to be involved throughout the study (e.g., suggestions with recruitment, sense-making, interpretation of the data, evaluation of the output) and to help with the dissemination of the findings. The dissemination of our findings will involve publication in scientific journals, social media outlets, webinars, electronic newsletters, presentations at conferences, as guided by our patient partners. We will adapt the content of our findings to health care professionals, researchers and people with CTS according to our PPIs suggestions.

## Discussion

Carpal tunnel syndrome is the most common entrapment neuropathy [27] but recruitment through secondary care could be challenging, especially when recruiting patients with mild carpal tunnel syndrome. Primary care facilities will be incorporated as needed to facilitate recruitment. Since our intervention and follow-up is significanly shorter than the current wait times (18 weeks at the time of the study in the UK) and the treatment provided is within the current clinical guidelines [1, 2], we expect this to be an incentive for potential eligible participants to be enrolled.

Neurodynamic exercises have shown effectiveness in the conservative management of people with entrapment neuropathies [14, 25] although their mechanisms of action are not fully understood. This study will explore the mechanisms of effect of neurodynamic exercises in detail, looking at their potential effect on microstructural nerve integrity, nerve function and inflammatory markers using carpal tunnel syndrome as a model system. Our findings are likely to help us understand which patients are likely to benefit from this treatment, an important first step in the progression towards precision physiotherapy.

### Electronic supplementary material

Below is the link to the electronic supplementary material.

Supplementary Material 1

## Data Availability

No datasets were generated or analysed during the current study.
