# Supplementary material for: Mechanisms of neurodynamic treatments (MONET): a protocol for a mechanistic, randomised, single-blind controlled trial in patients with carpal tunnel syndrome
Source: BMC Musculoskelet Disord. 2024 Jul 27;25:590. doi: 10.1186/s12891-024-07713-6 (PMC11282828; doi:10.1186/s12891-024-07713-6)
Supplement: Supplementary file 1 — Supplementary Material 1 [file 12891_2024_7713_MOESM1_ESM.docx]

**SUPPLEMENTARY INFORMATION**

**Suppl. Table 1.** MRI sequence parameters at the cervical spine

|  | **T2 SPACE STIR** | **RESOLVE (DWI)** |
| --- | --- | --- |
| Repetition time (TR) | 3500 ms | 8390 ms |
| Echo time (TE) | 164 ms | 44 ms, 68ms |
| Flip angle (FA) | N/A | 90° and 180° |
| Field of view (FoV) | 192 mm | 140 mm |
| Dimensionality | 3D | 2D |
| Slice thickness | 1mm | 1.5mm |
| Number of slices | 112 | 70 |
| In-plane resolution | 1x1mm | 1.5x1.5mm |
| Echo spacing | 4.32ms | 0.36ms |
| Fat suppression | None | Fat saturation |
| Bandwidth | 457Hz/Px | 1087 Hz/Px |
| No. gradient directions | N/A | 6 |
| Readout segments | N/A | 5 |
| Scan time single phase-encode blip (right-left or left-right) | N/A | 5.52 |
| Total scan time (min) | 5.43 | 6.02 |

N/A: not applicable.

**APPENDIX A:**

**A. Leaflet for participant allocated to the neurodynamic exercise group**

**Carpal Tunnel Syndrome**

**What is it?**

Carpal Tunnel Syndrome (CTS) is a condition caused by the median nerve being compressed as it passes though the carpal tunnel in the wrist. The carpal tunnel is narrow and is formed by the wrist bones and a thick ligament, as shown in the picture below. CTS affects more women than men and is most common in those aged between 40 and 65.


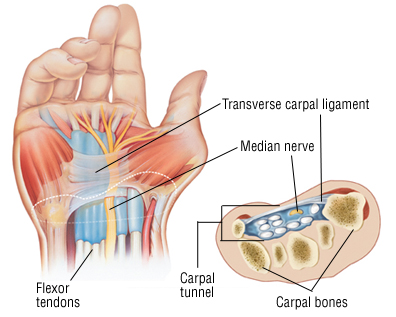


http://www.drugs.com/health-guide/images/205264.jpg

CTS can cause a variety of symptoms, including pain, tingling, numbness, swelling, weakness or clumsiness of the thumb, index, middle and ring fingers. Symptoms are often worse overnight, but may also occur throughout the day. Any condition that reduces the amount of space within the carpal tunnel can cause CTS. Examples of these can include (but are not limited to): inflammation, wrist arthritis, wrist fractures, fluid retention, diabetes or rheumatoid arthritis.

**Neurodynamic exercises**

You have been allocated to the neurodynamic exercise group. Neurodynamic exercises are designed to gently move your nerve and tendons in the carpal tunnel without putting strain on them. These neurodynamic exercises have been shown to reduce inflammation and improve regeneration of the nerve in preclinical studies. Our research team has shown that they reduce the need for carpal tunnel surgery in patients who are already listed to undergo carpal tunnel surgery.

We ask you to complete **8 exercises 6 times a day for the next 6 weeks**. One exercise session will only take a couple of minutes. The exercises are designed to gently move the tendons and the nerve at the wrist. This may reduce the swelling. You will basically pump the swelling out of your carpal tunnel!

It is **very important to perform the exercises frequently** throughout the day (minimum 5 times). Every time, you pump a little bit of swelling away. Exercise when you are waiting for the kettle to boil, when you are in the shower, when you are waiting at the check-out or waiting for the bus, when you are watching TV, during short breaks at work, or any other moment that is convenient.

One session per hour is enough though. There is no need to do more. We do not want you to end up with overuse symptoms.

If you exercise, please **perform all 8 exercises in 1 session** and then wait 1 hour before you do it again. You should not experience any discomfort or pain during or after the exercises. Should you encounter any difficulties with any of these exercises, please call 01865234821.

These are the 8 exercises that you will perform during the next 6 weeks. Please keep in mind that the first two exercises will evolve to more advanced sequences as you progress with your sessions. This way we can better pump out the swelling from your nerve.

It is important that you do not start the more advanced sequences before the suggested time. Your nerve needs some time to get ready to these progressions. If during the progression you experience any discomfort of pain, please contact ask.monet@ndcn.ox.ac.uk.

**WEEK 1 AND 2**

1. **Nerve Glide: ‘waiter’**

| 1. Hold your arm close to your body and forearm out to the side with your palm facing up and wrist bent back (as if you have a glass of water on your hand) 2. Straighten the elbow and hand ¾ of the way (remember to keep the wrist in a position not to drop your glass) 3. Alternate these positions. 4. Repeat **10** times, 6 times per day. | 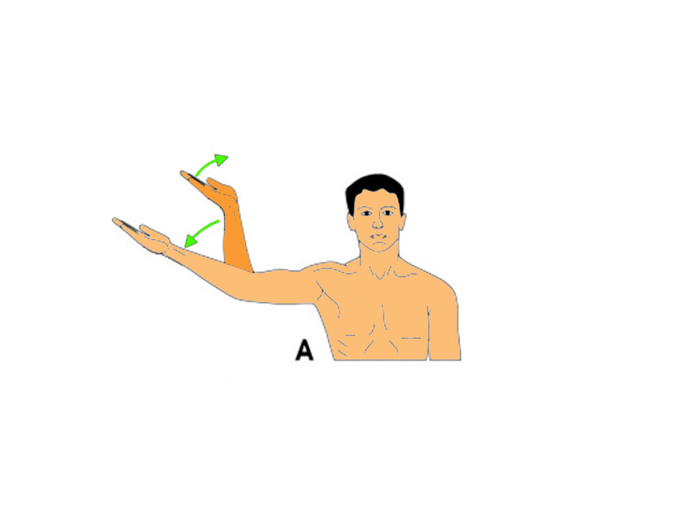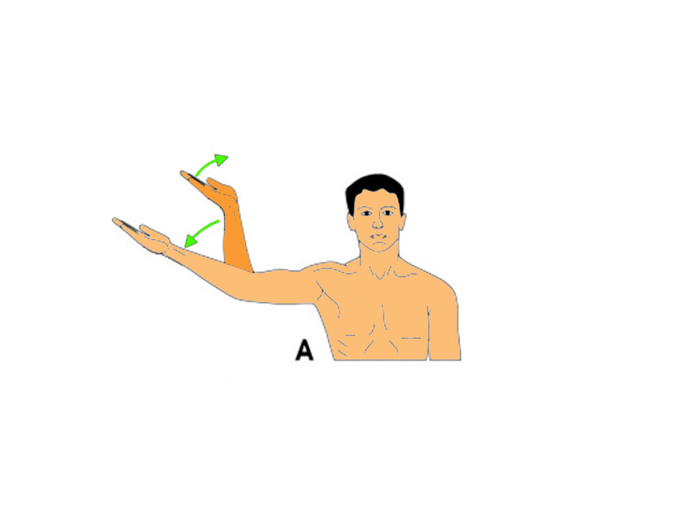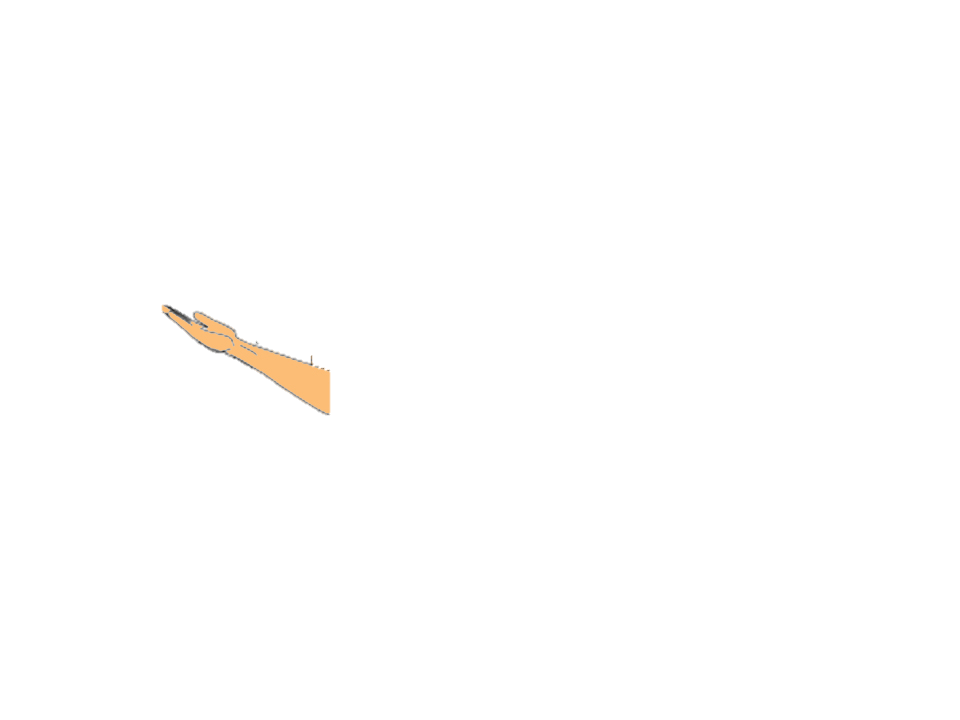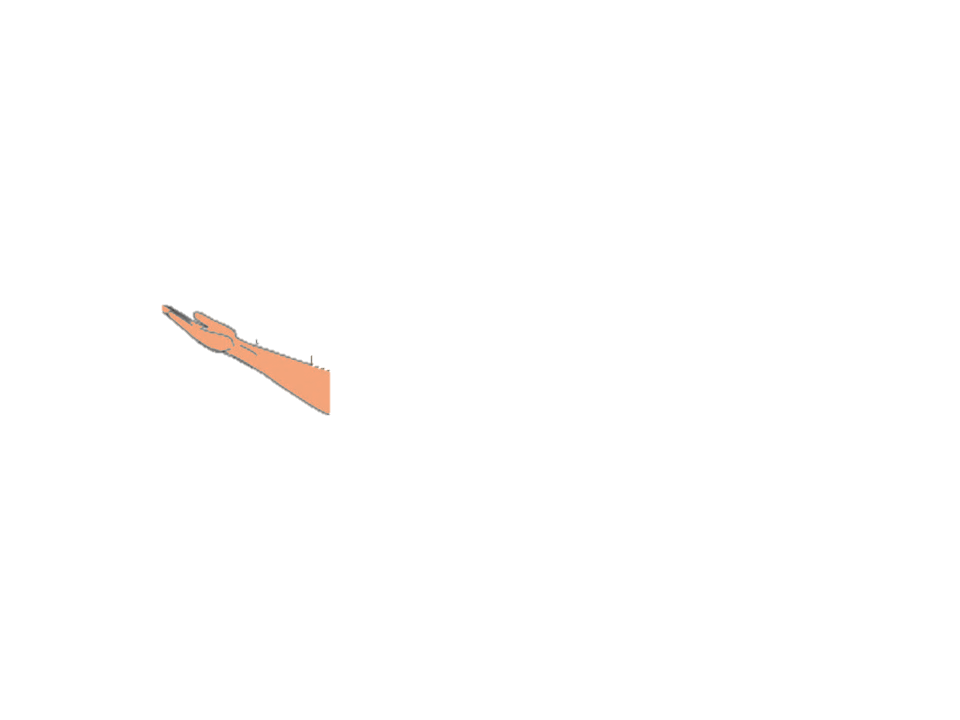 |
| --- | --- |

1. **Nerve Tensioner: ‘drop the ball’**

| 1. Hold your arm close to your body and forearm out to the side with your palm facing up and wrist straight  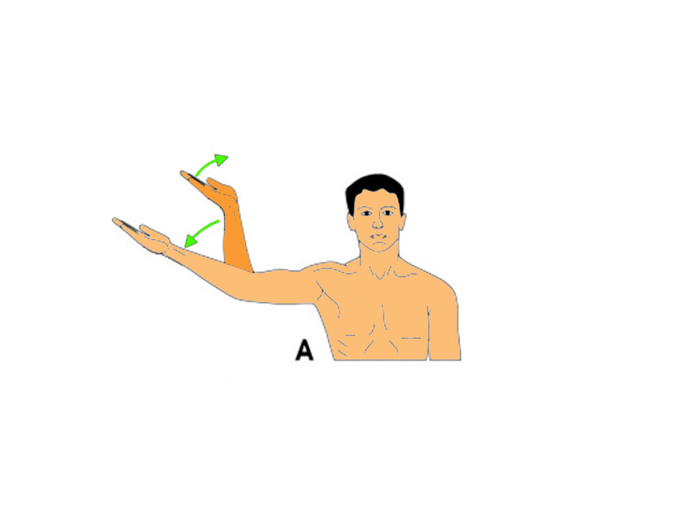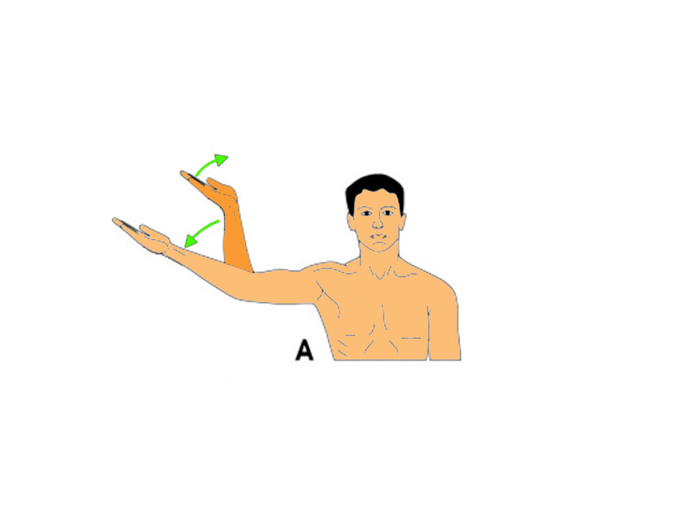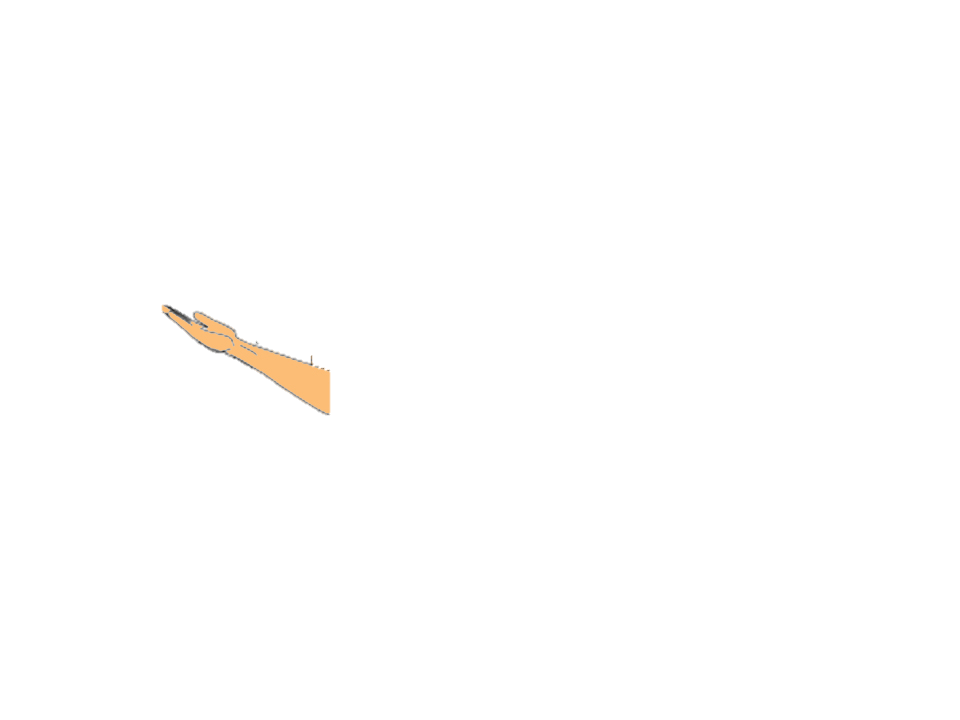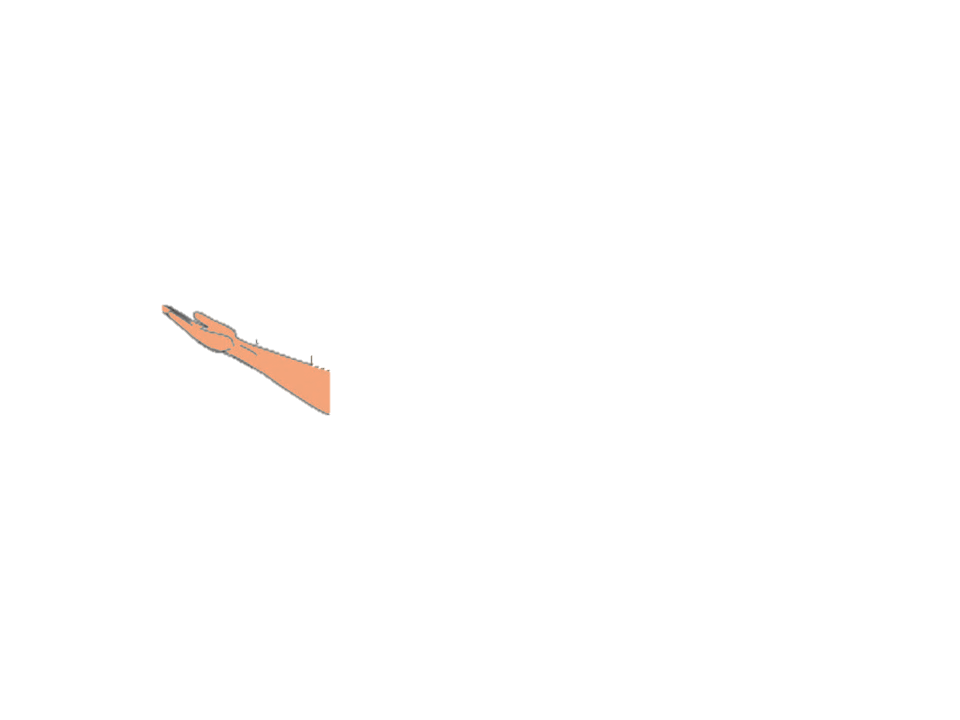  1. Straighten the elbow ¾ of the way and bend the wrist back 2. Alternate these positions. 3. Repeat **10** times, 6 times per day. |  |
| --- | --- |

1. **‘Shoulder circles’**

| 1. Circle your shoulders with big movements (up, back, down, forward) 2. Repeat **10** times, 6 times per day | 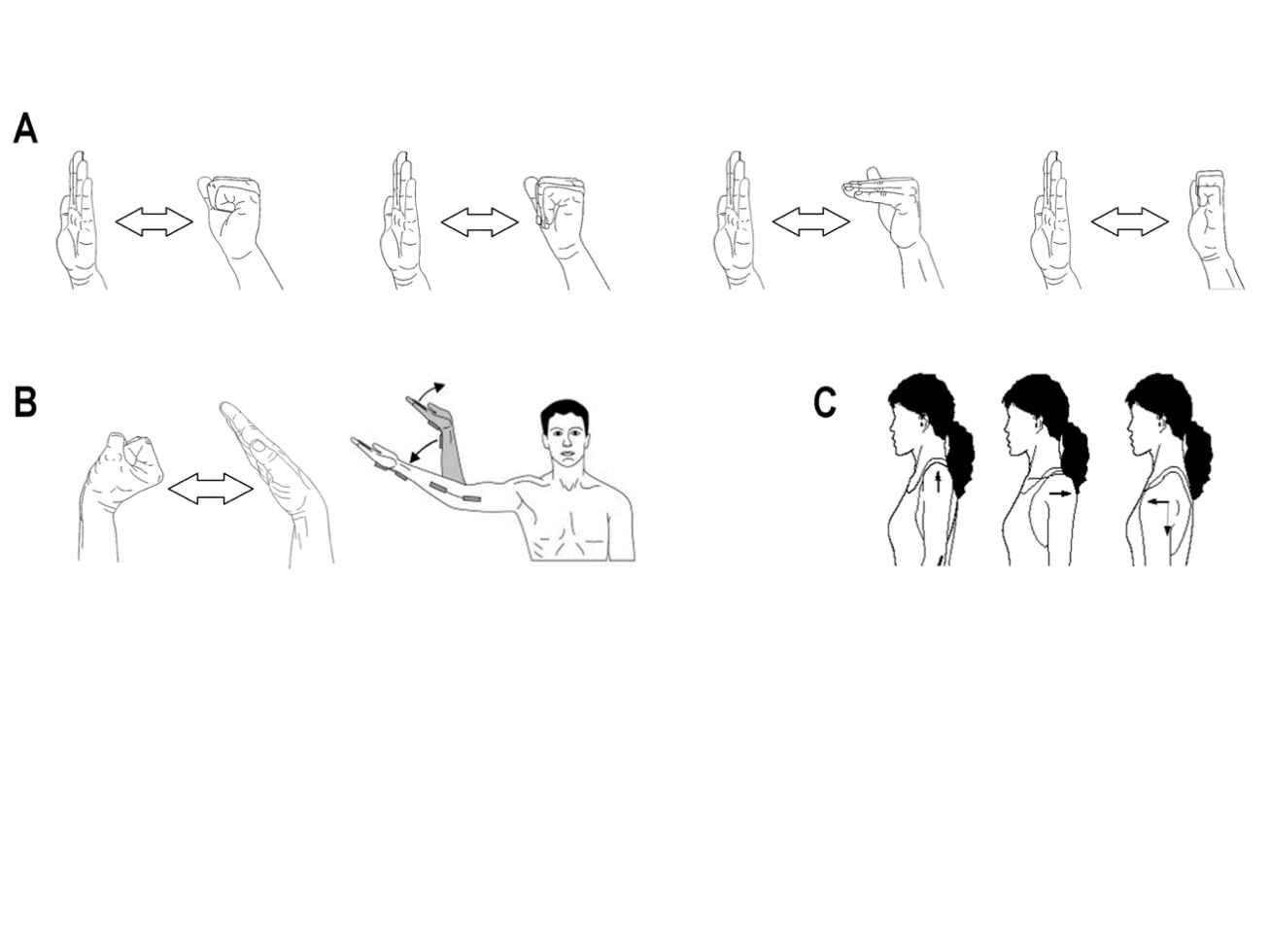 |
| --- | --- |

1. **Nerve Glide: ‘throw and catch a ball’**

| 1. Bend your wrist forward (flexion) and back (extension). Keep your fingers relaxed. 2. Keep your elbow still while you do the exercises. 3. Alternate these positions. 4. Repeat **10** times, 6 times per day. | **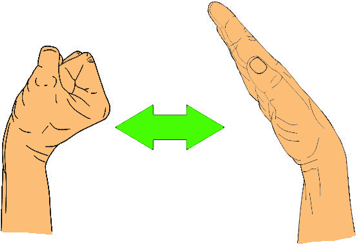** |
| --- | --- |

1. **Tendon Glide: ‘fist’**

| 1. Start with your fingers and wrist straight. 2. Make a fist 3. Alternate these positions. 4. Repeat **10** times, 6 times per day. |  |
| --- | --- |

1. **Tendon Glide: ‘fist with straight fingers’**

| 1. Start with your fingers and wrist straight. 2. Make a fist with straight fingers 3. Alternate these positions. 4. Repeat **10** times, 6 times per day. |  |
| --- | --- |

1. **Tendon Glide: ‘table top’**

| 1. Start with your fingers and wrist straight. 2. Move your fingers into a ‘table top position as per picture on the right side 3. Alternate these positions. 4. Repeat **10** times, 6 times per day. |  |
| --- | --- |

1. **Tendon Glide: ‘monkey grip’**

| 1. Start with your fingers and wrist straight. 2. Move your fingers into a monkey grip position as per picture on the right side 3. Alternate these positions. 4. Repeat **10** times, 6 times per day. |  |
| --- | --- |

**WEEK 3 AND 4**

**1b. Nerve Glide: ‘waiter’**

| 1. Hold your arm out to side with your palm facing up and wrist bent back (as if you have a glass of water on your hand) 2. Bend your elbow and wrist to bring your hand closer to your body (imaging you have a glass of water on your hand) 3. Alternate these positions. 4. Repeat **10** times, 6 times per day. | 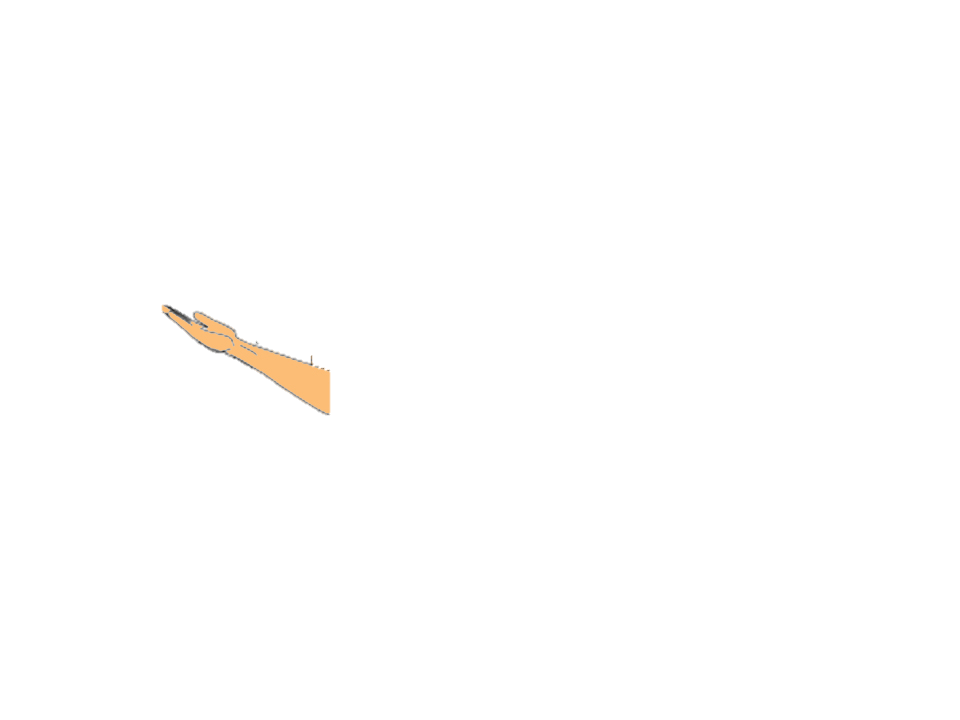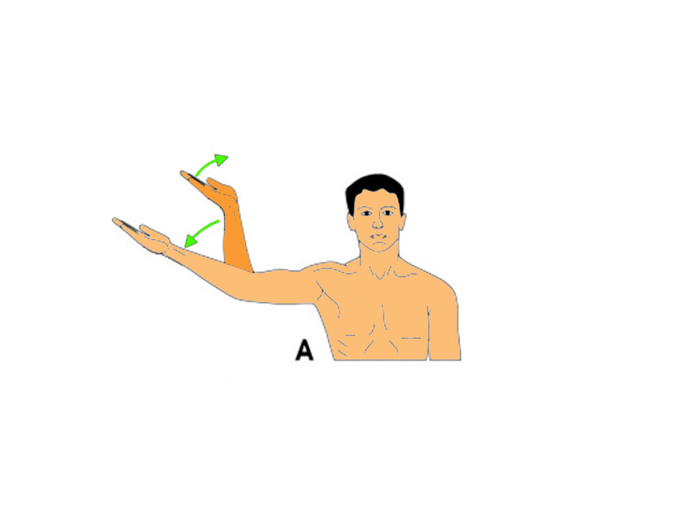 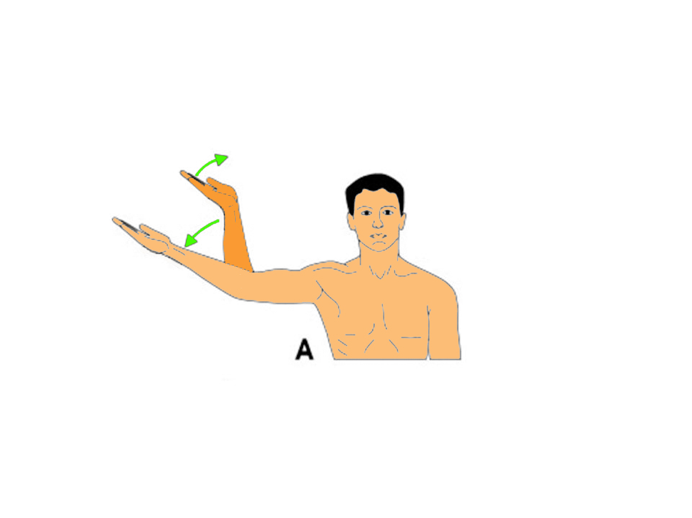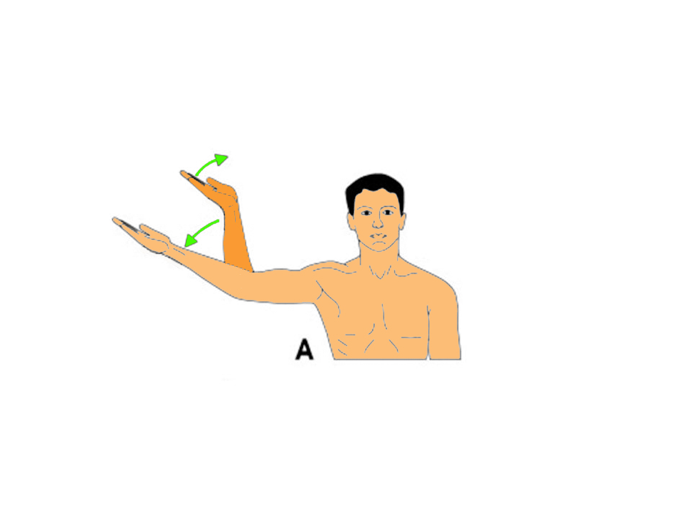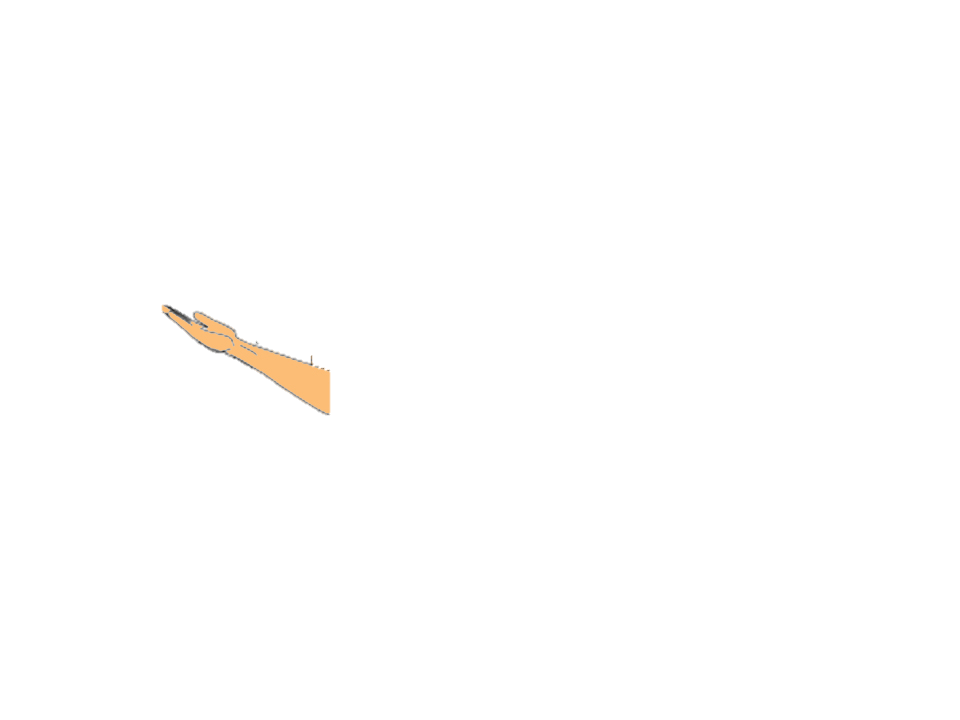 |
| --- | --- |

**2b. Nerve Tensioner: ‘drop the ball’**

| 1. Hold your arm straight and out to side with your palm facing up and wrist in line with the arm 2. Move your wrist and fingers back, as if you were dropping a ball from your hand 3. Alternate these positions. 4. Repeat **10** times, 6 times per day. | 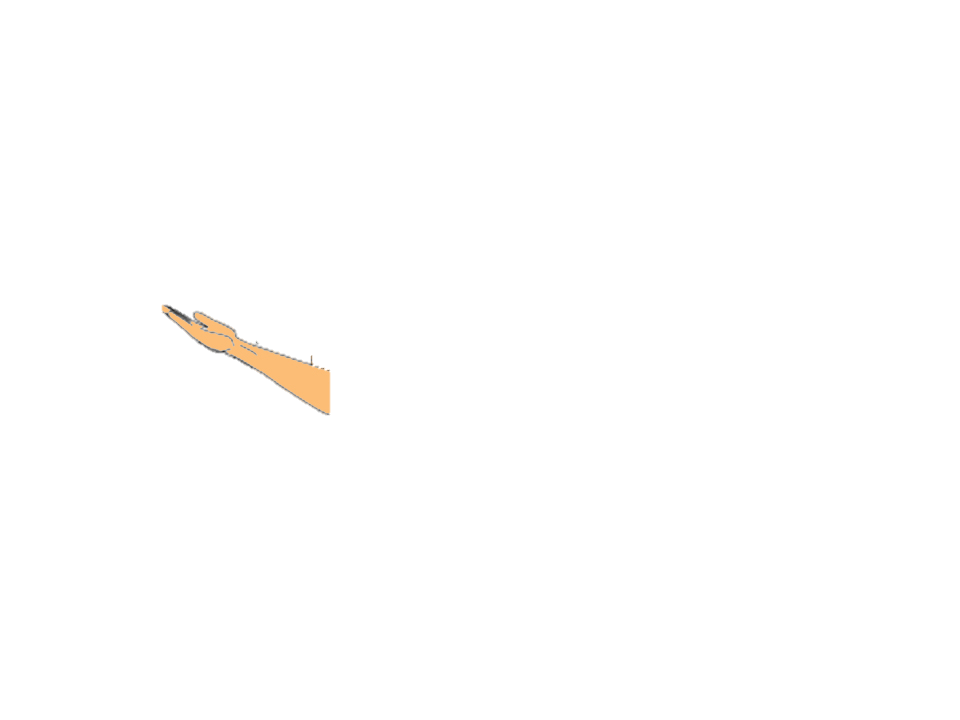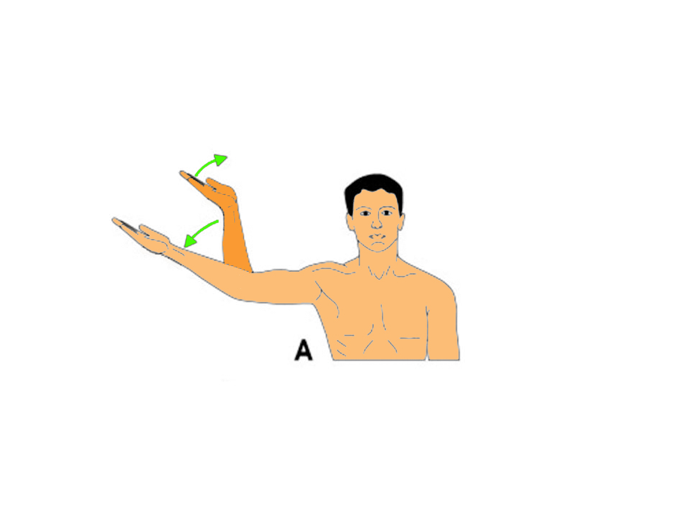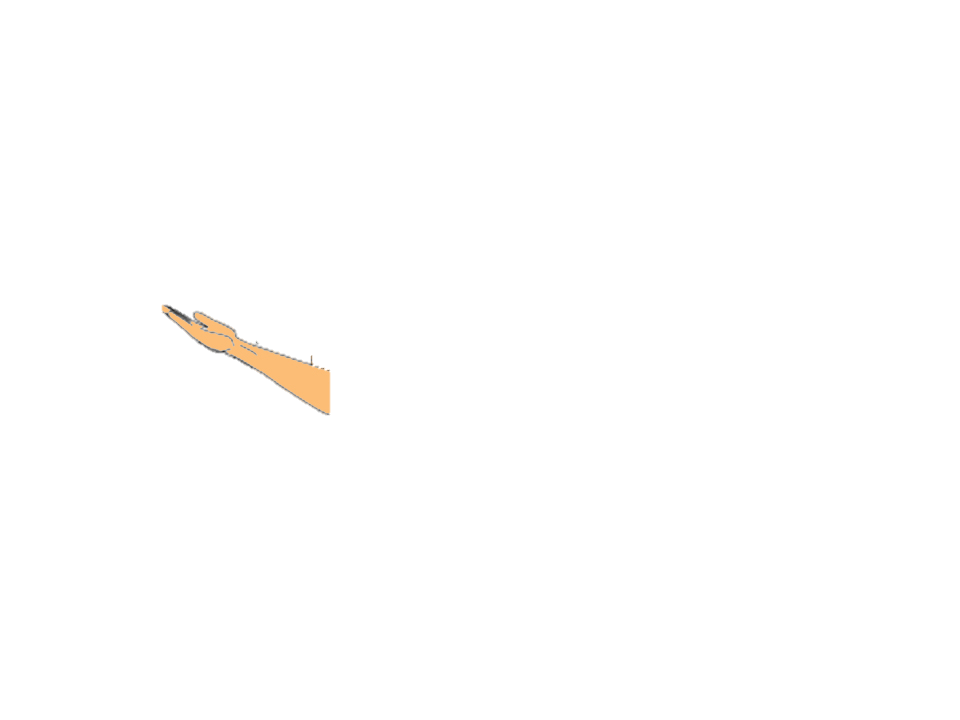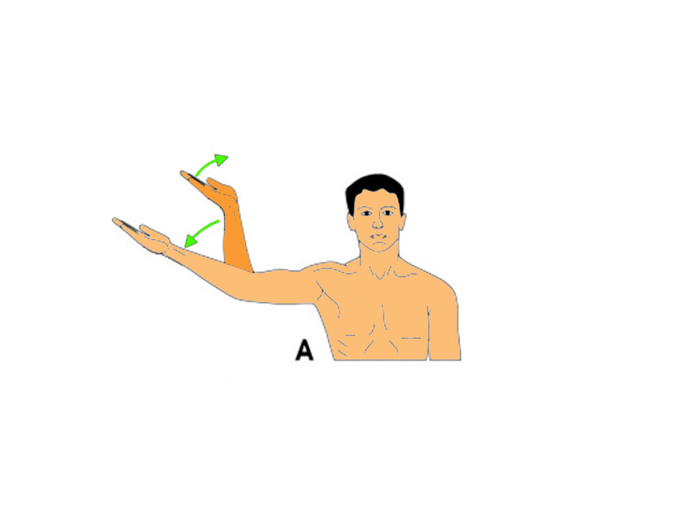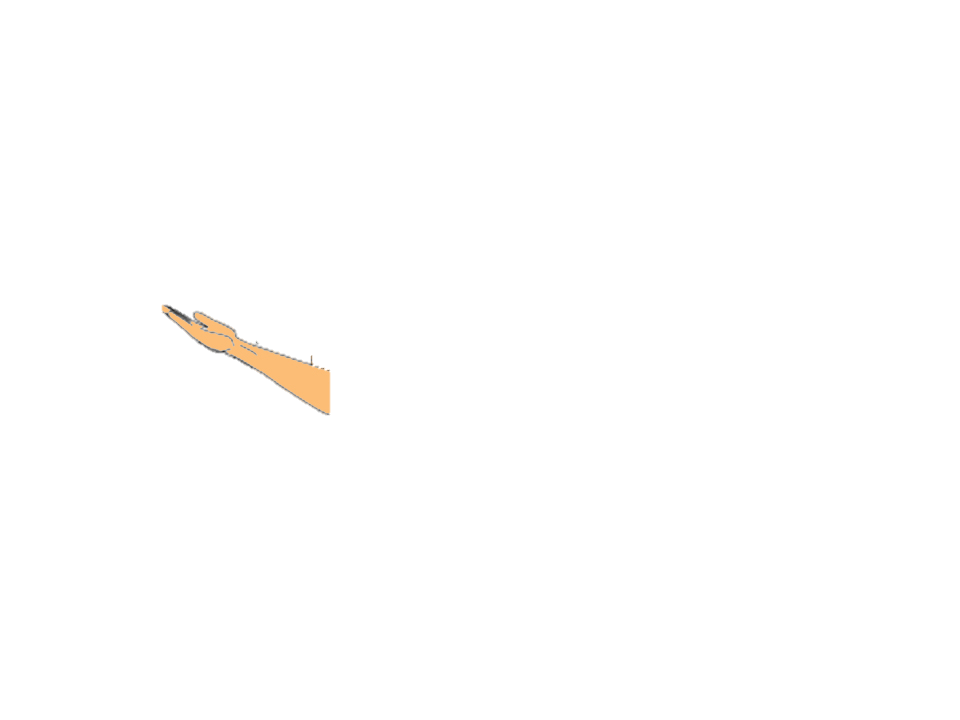 |
| --- | --- |

Now you can continue with exercises 3-8 from week 1-2.

**WEEK 5 AND 6**

For the progression of exercises 1 and 2 we will practice some head movements first. Bring the ear closer to your shoulder keeping your sight and nose pointing to the front, then, slowly, bring back the head to the original vertical position. Repeat this movement until you feel comfortable doing it. Please check that your shoulders are relaxed, only your head is moving.


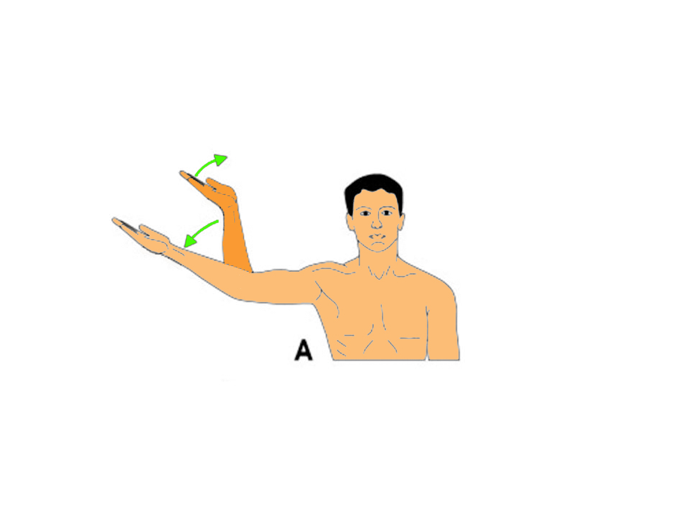

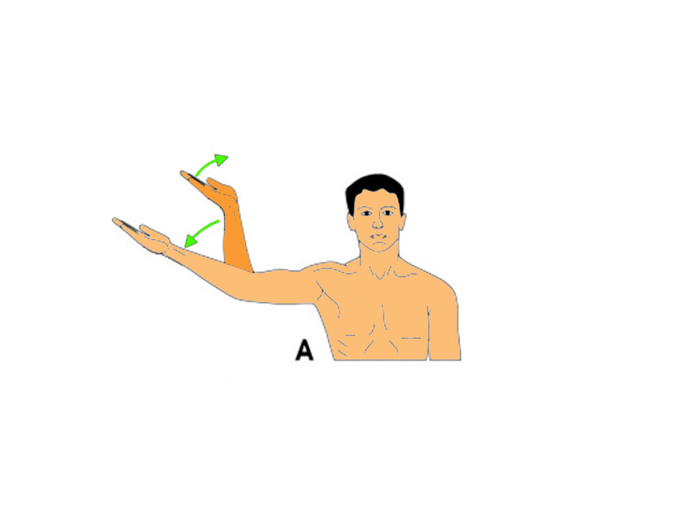

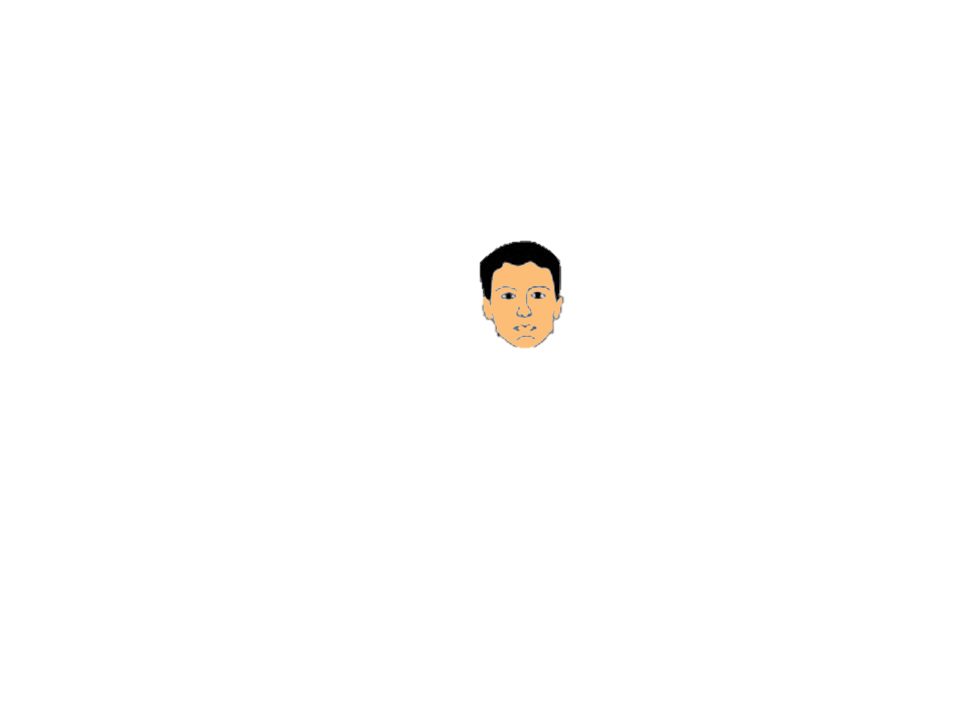

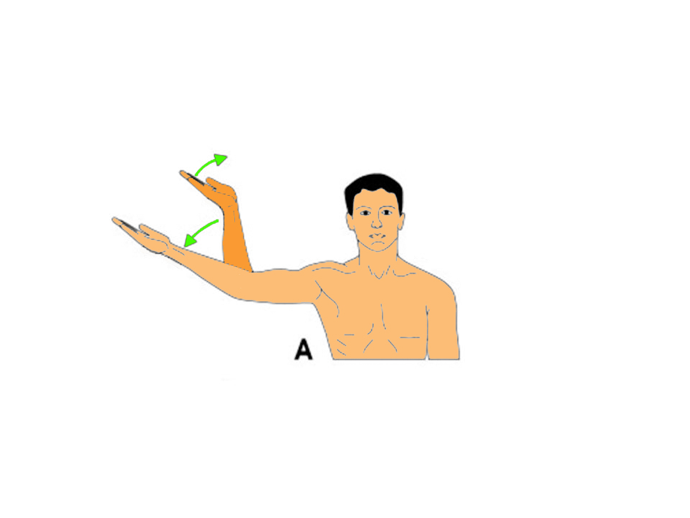

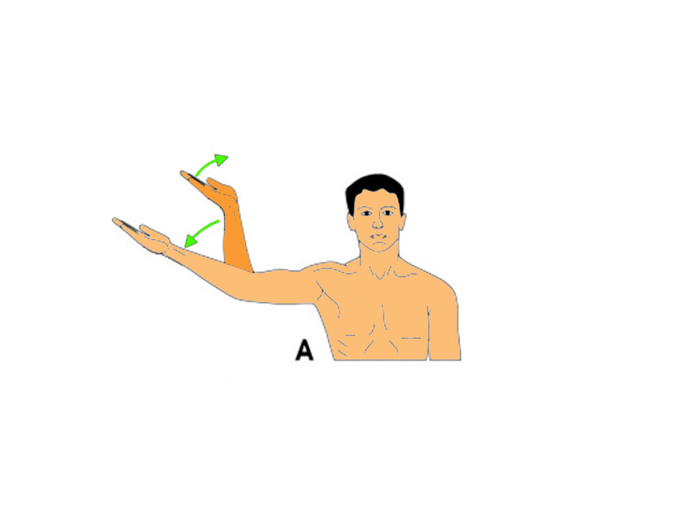


**1c. Nerve Glide: ‘waiter’**

| 1. Hold your arm out to side with your palm facing up and wrist bent back (as if you have a glass of water on your hand). Keep your head in line with the rest of your body. 2. While you bend your elbow and wrist to bring your hand closer to your body (imaging you have a glass of water on your hand), bring the opposite ear closer to the opposite shoulder. Remember to keep your nose always pointing to the front while moving your head. 3. Alternate these positions. 4. Repeat **10** times, 6 times per day. | 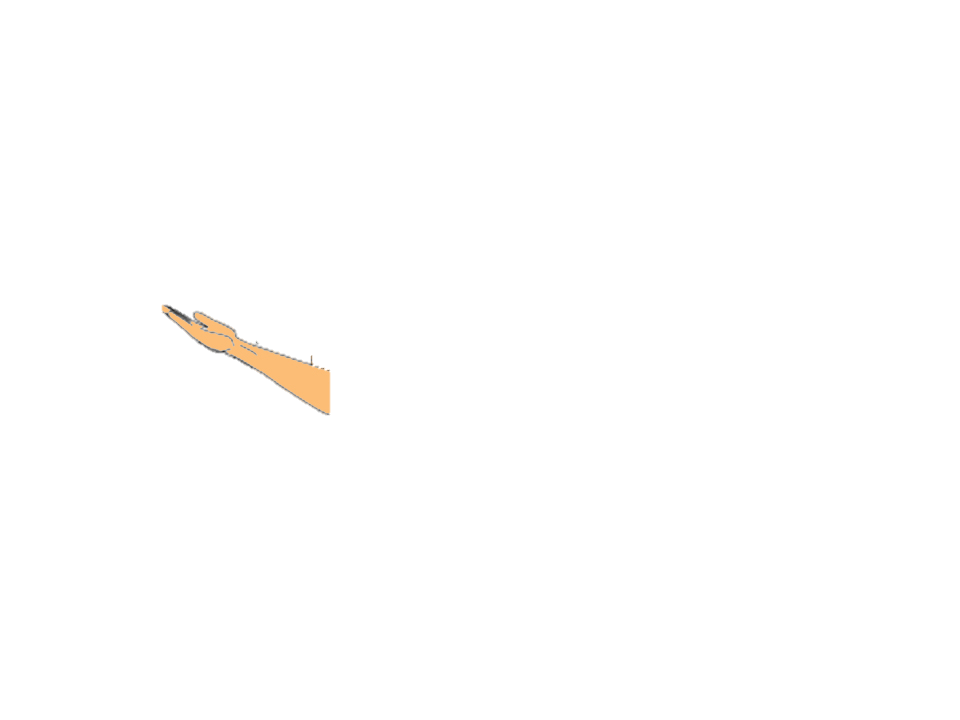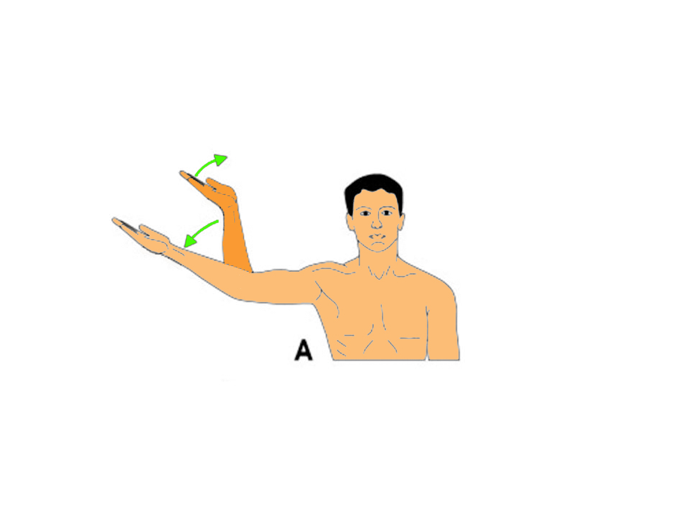 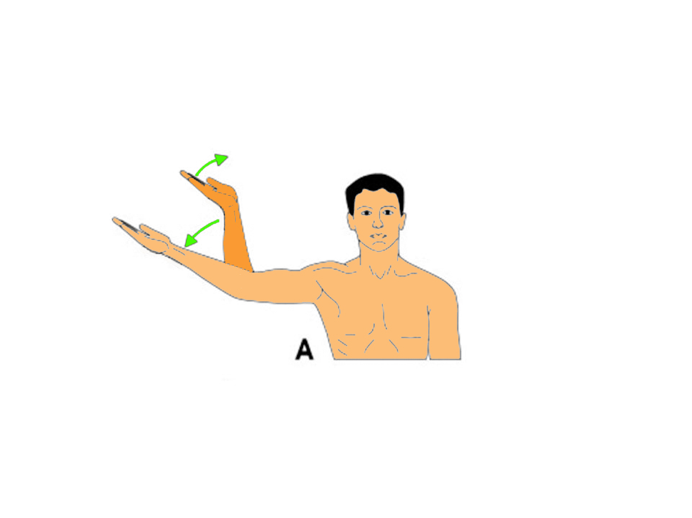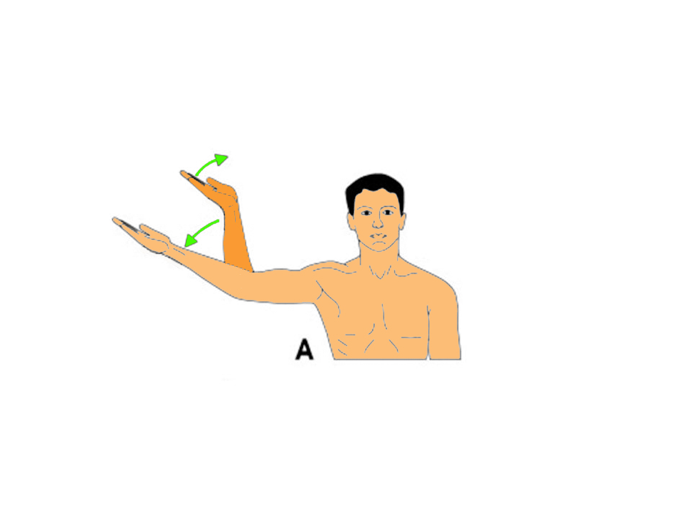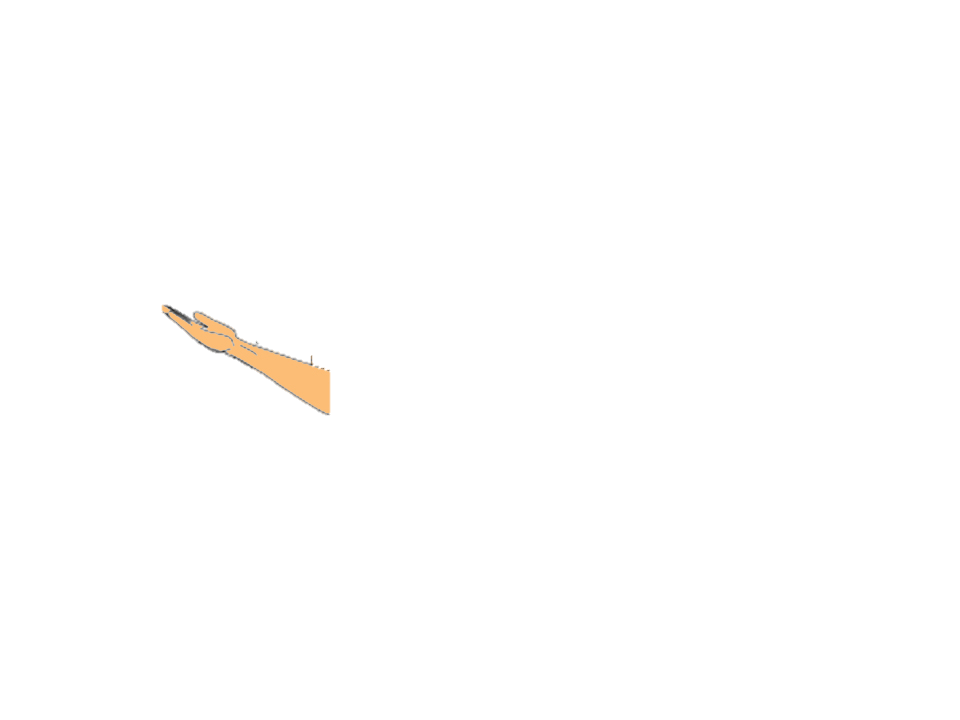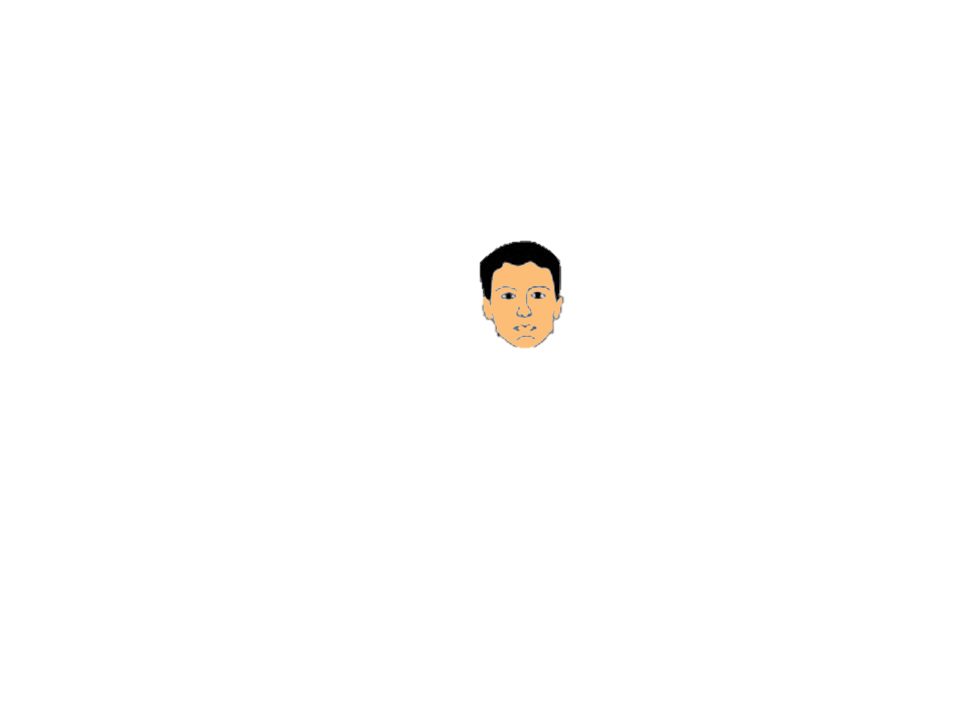 |
| --- | --- |

**2c. Nerve Tensioner: ‘drop the ball’**

| 1. Hold your arm straight and out to side with your palm facing up and wrist in line with the arm 2. While you move your wrist and fingers back, as you were dropping a ball from your hand, bring the opposite ear closer to the opposite shoulder. Remember to keep your nose always pointing to the front while moving your head. 3. Alternate these positions. 4. Repeat **10** times, 6 times per day. | 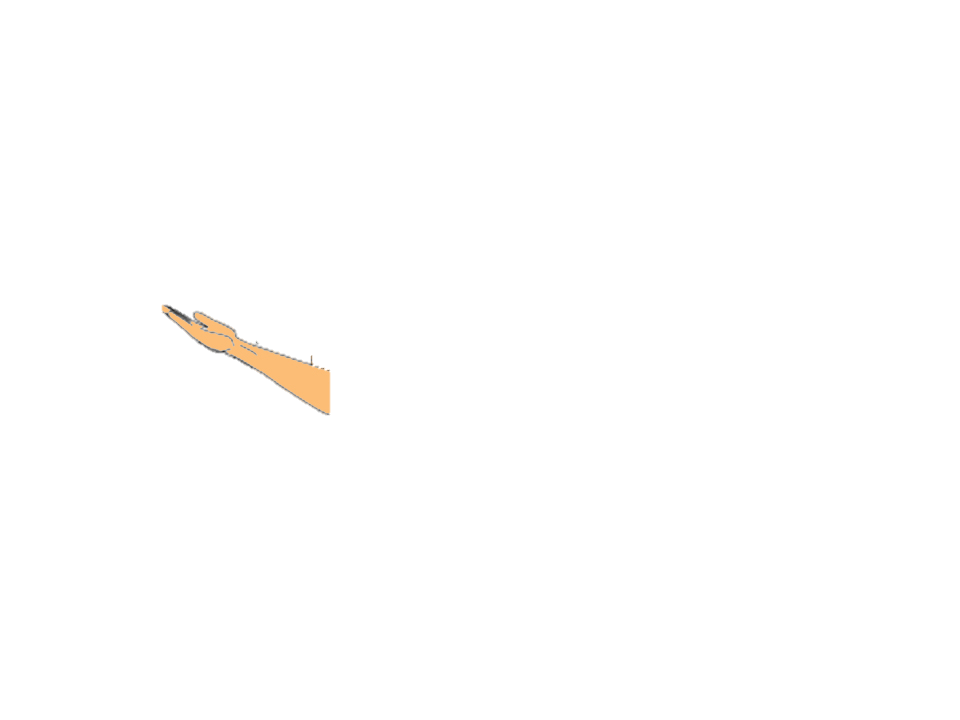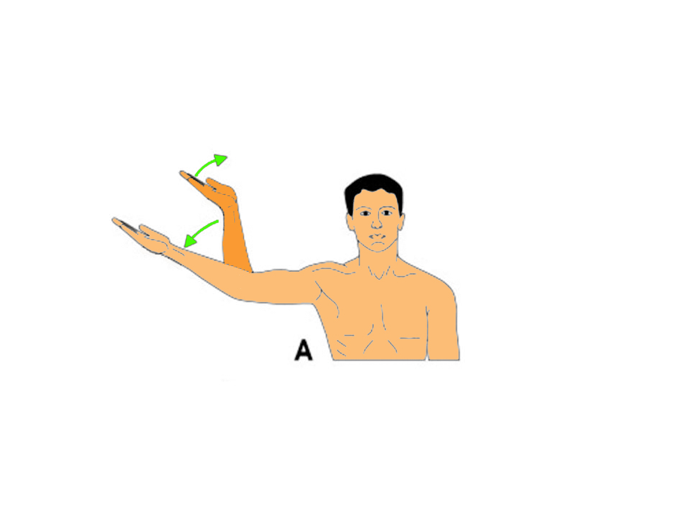 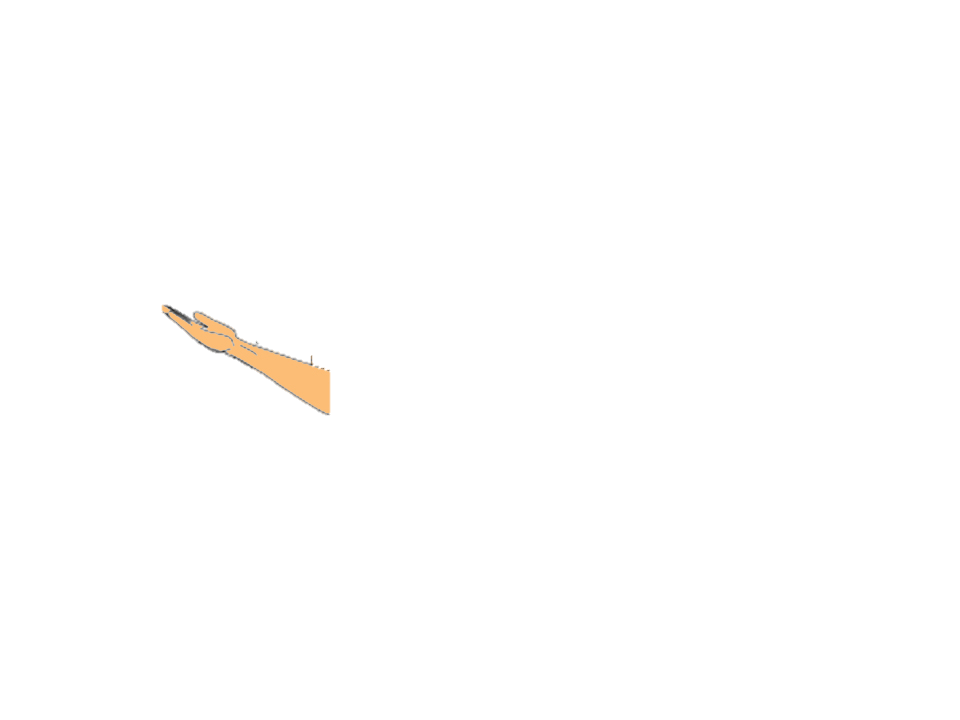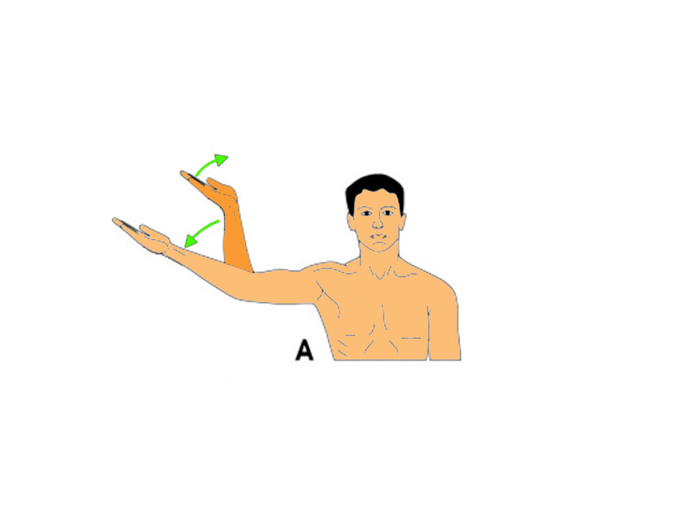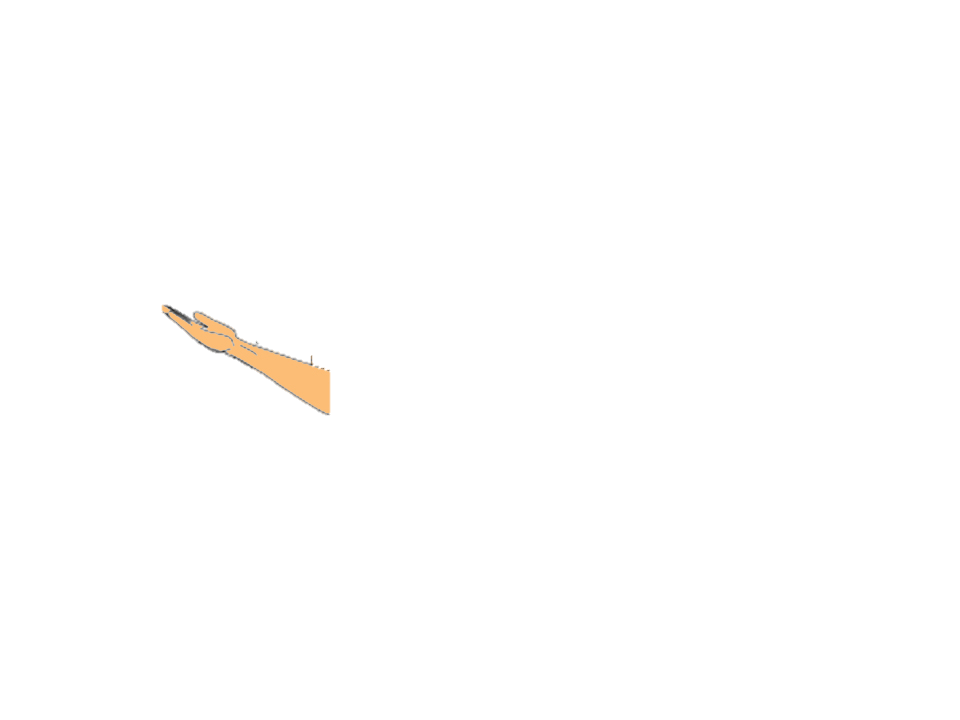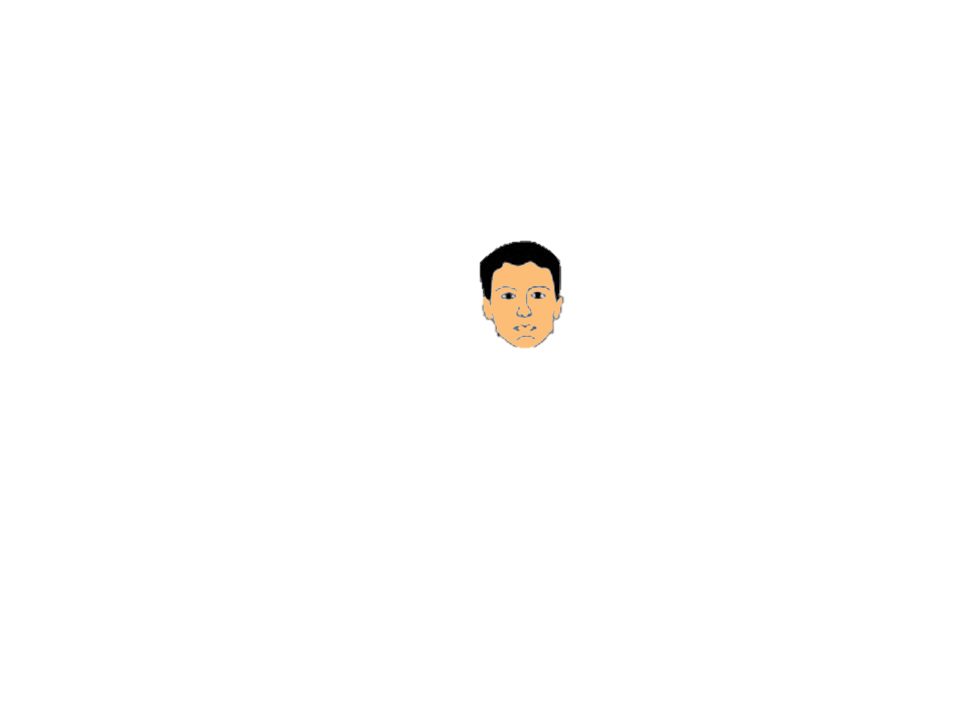 |
| --- | --- |

You can find a video demonstrating the individual exercises and the complete sequences with the progression in a link on your mailbox.

| **IMPORTANT:**   - Perform 1 repetition per second. Opening and closing the hand is considered 1 repetition. - Do not squeeze. It is all about movement, not strength. Do the exercises gently, but reasonably quickly. - Perform **10 repetitions of each exercise per session**. - Perform at least **6 sessions per day** - **Distribute the sessions evenly** over the morning, afternoon and evening, leaving 1 hour between sessions - If you feel an increase in your pain or symptoms you should stop that exercise and contact the study team on <email> or <phone> - **Please do not start any other treatments or new exercises for carpal tunnel syndrome during the 6 week intervention period.** |
| --- |

**WEEK 1-2**

**Neurodynamic exercises summary: take me everywhere!**
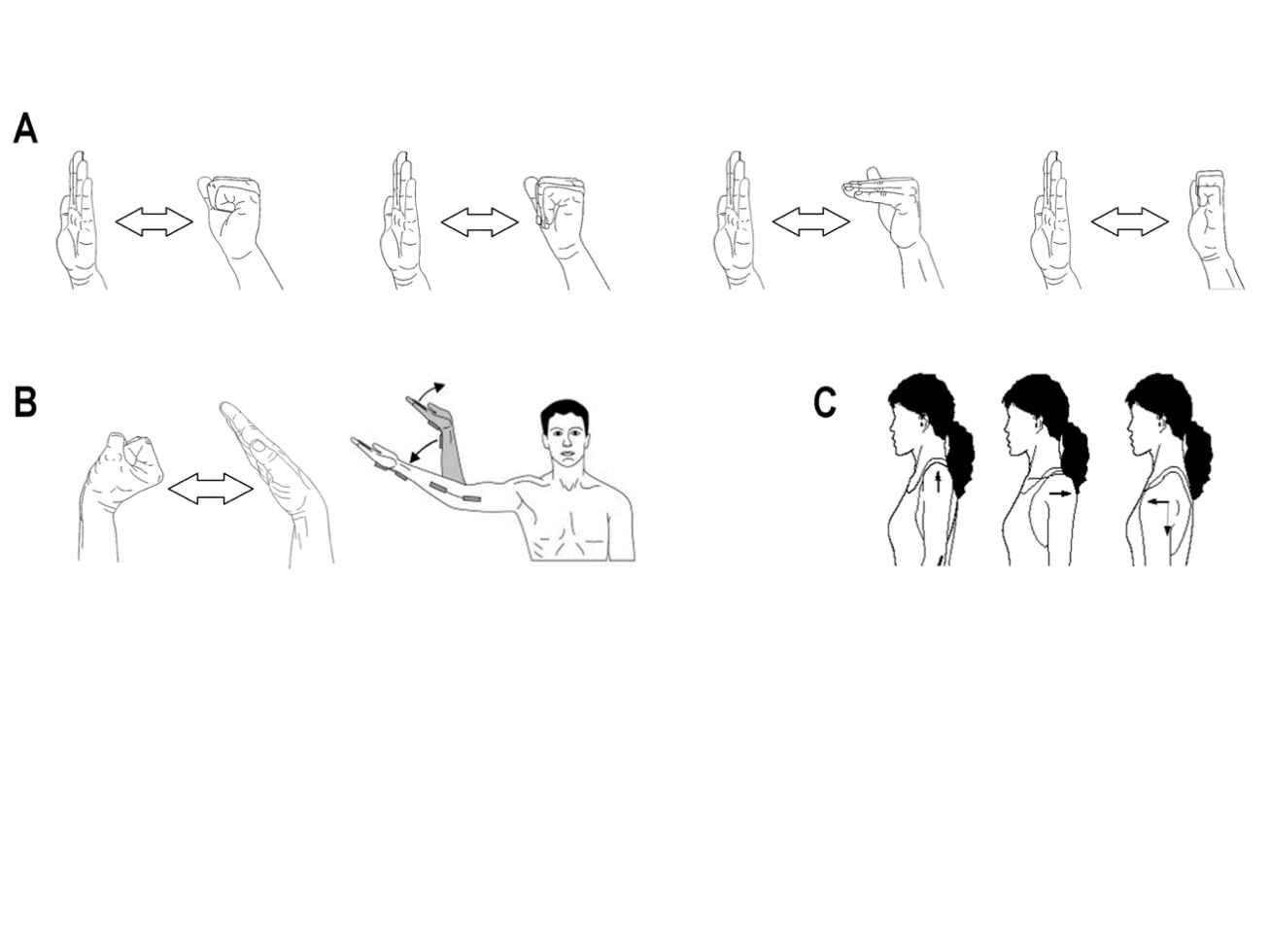

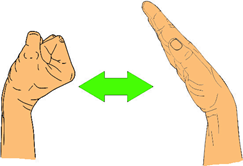

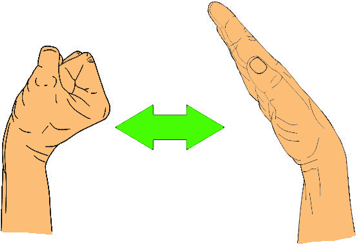


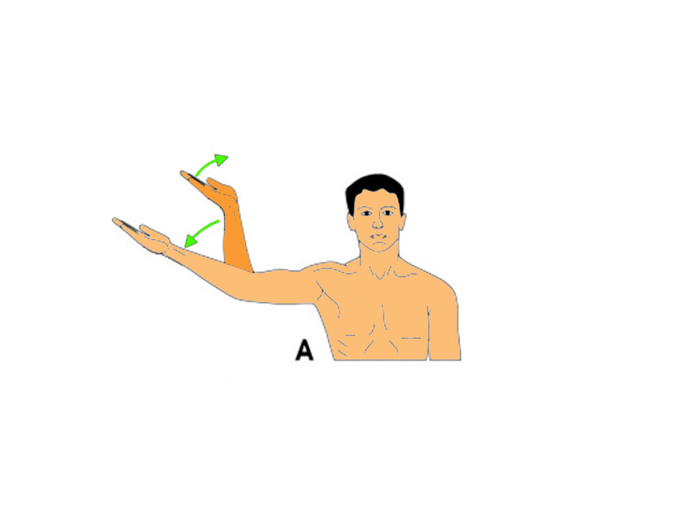

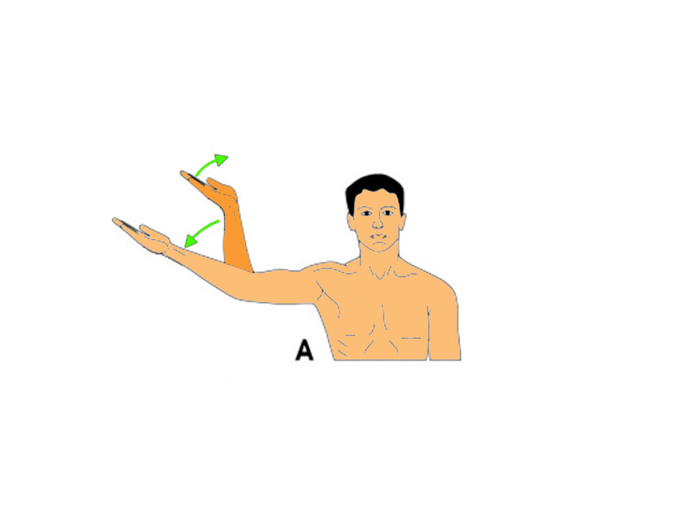

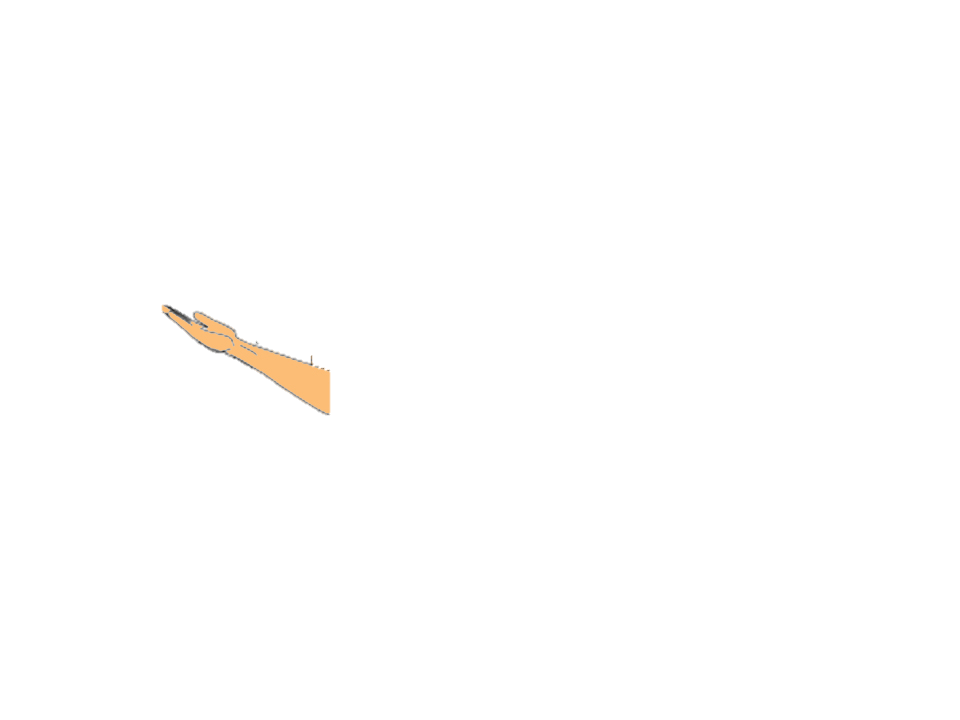

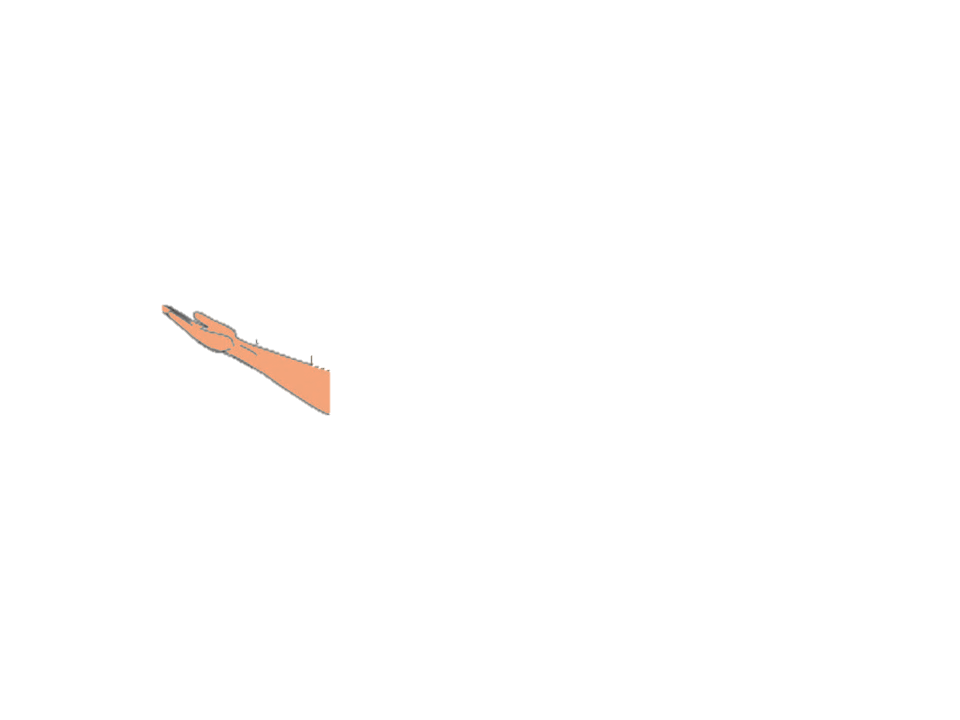


**EXERCISE DIRECTIONS: SUMMARY**

**How many?**

10 repetitions of each exercise per session.

**How often?**

6 sessions/day. Distribute the sessions evenly over the morning, afternoon and evening, leaving 1 hour between sessions.

**How fast?**

1 repetition/second (opening and closing hand is 1 rep).

**How gentle?**

It is all about movement, not strength. Do the exercises gently, but reasonably quickly.

**If you feel an increase in your pain or symptoms you should stop that exercise and contact us!**

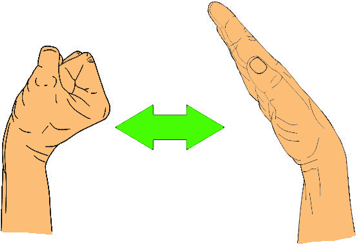


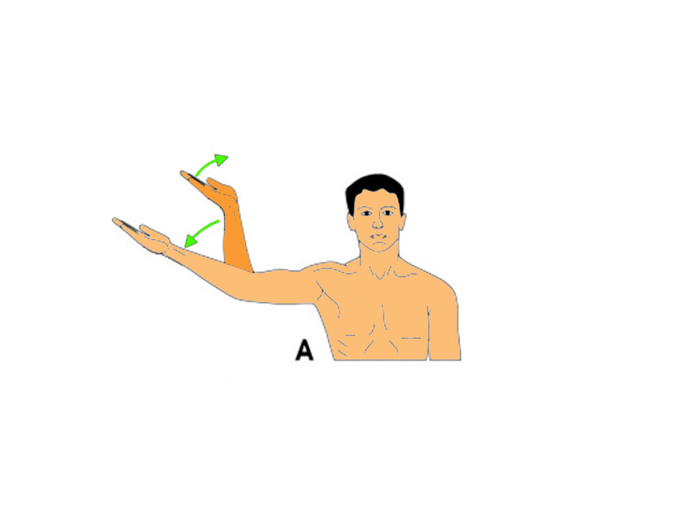

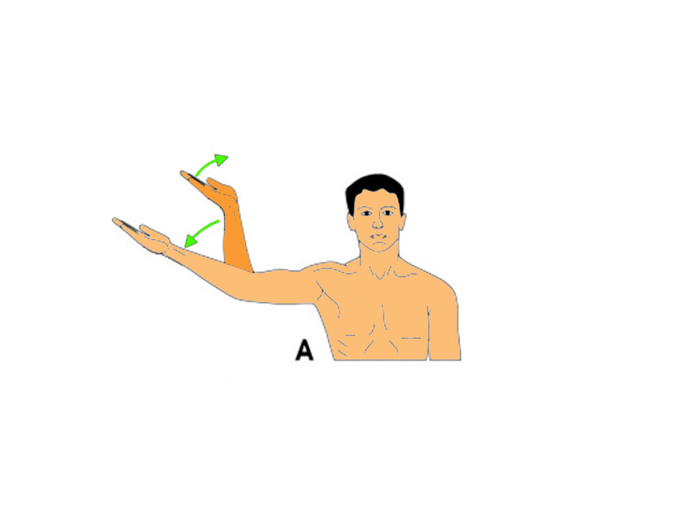

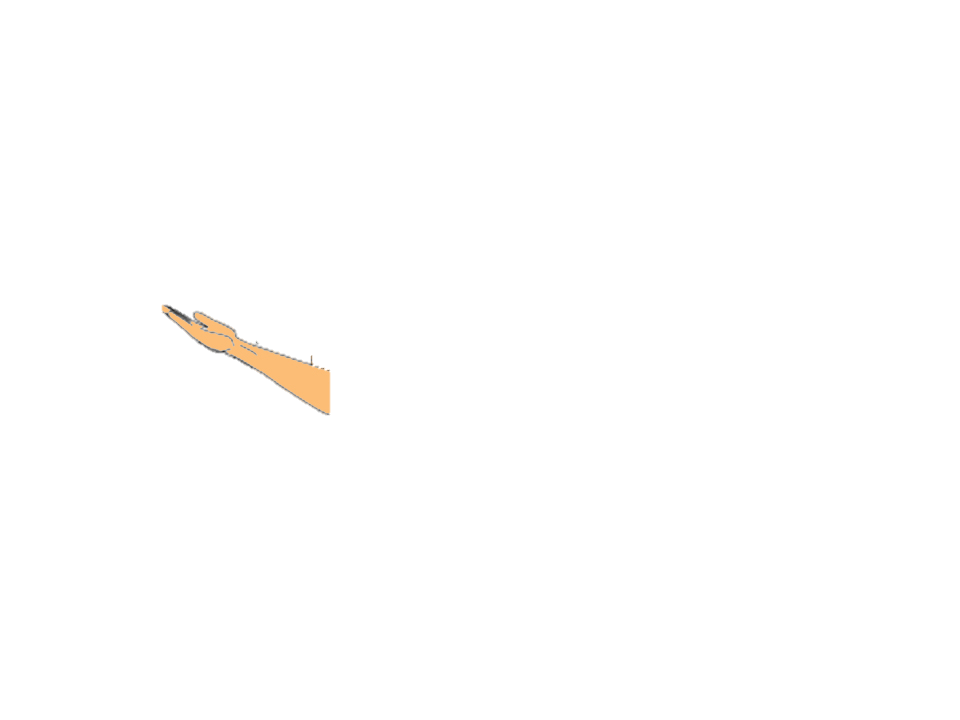

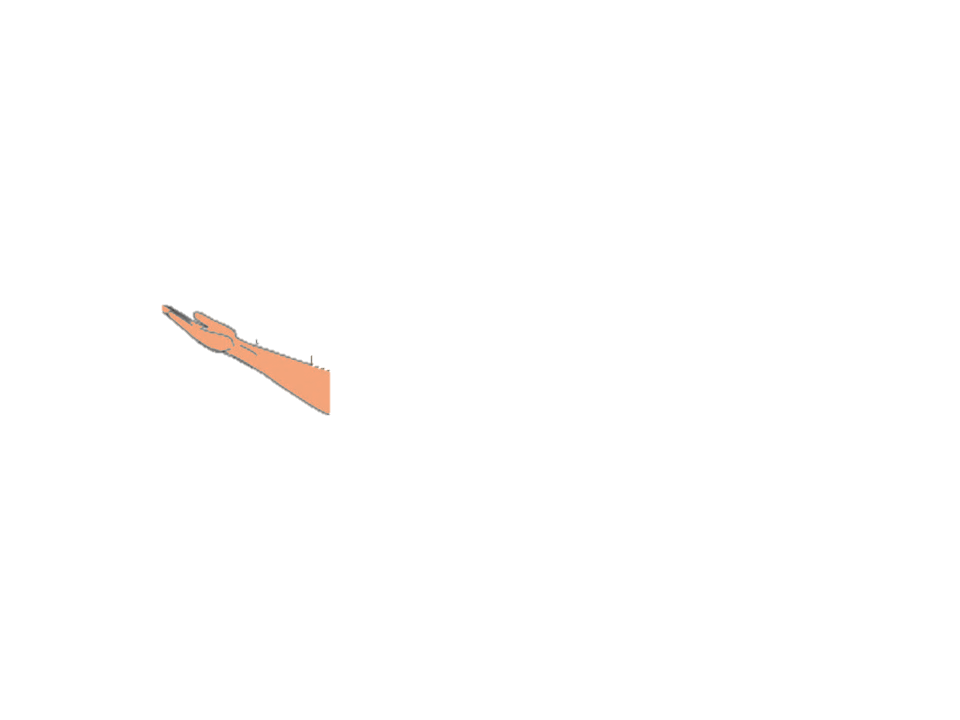


**4. Nerve Glide: ‘throw and catch a ball’**

**3. Shoulder circles**

**2. Nerve Tensioner: ‘drop the ball’**

**1. Nerve Glide: ‘waiter’**

**8. Tendon Glide: ‘monkey grip’**

**7. Tendon Glide: ‘table top’**

**6. Tendon Glide: fist with straight fingers**

**5. Tendon Glide: fist**

**WEEK 3-4**

**Neurodynamic exercises summary: take me everywhere!**
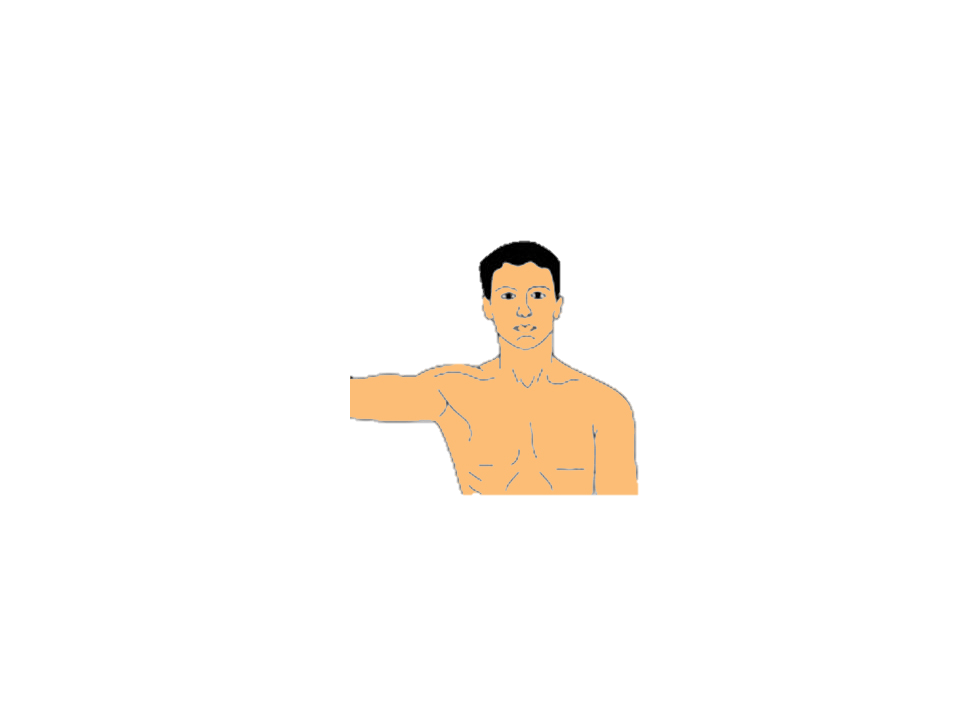

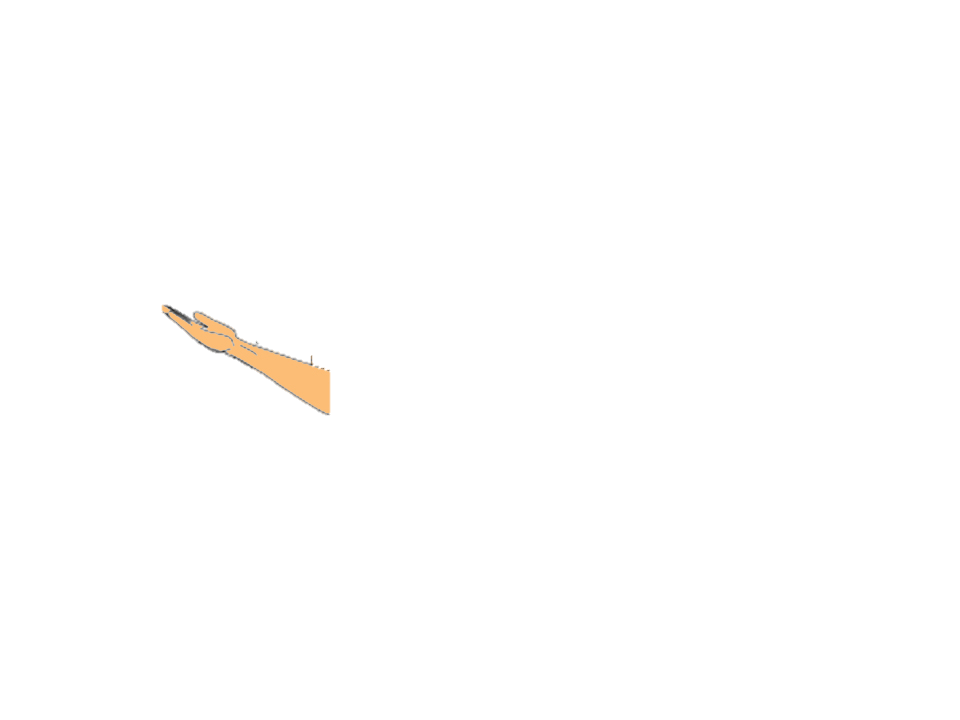

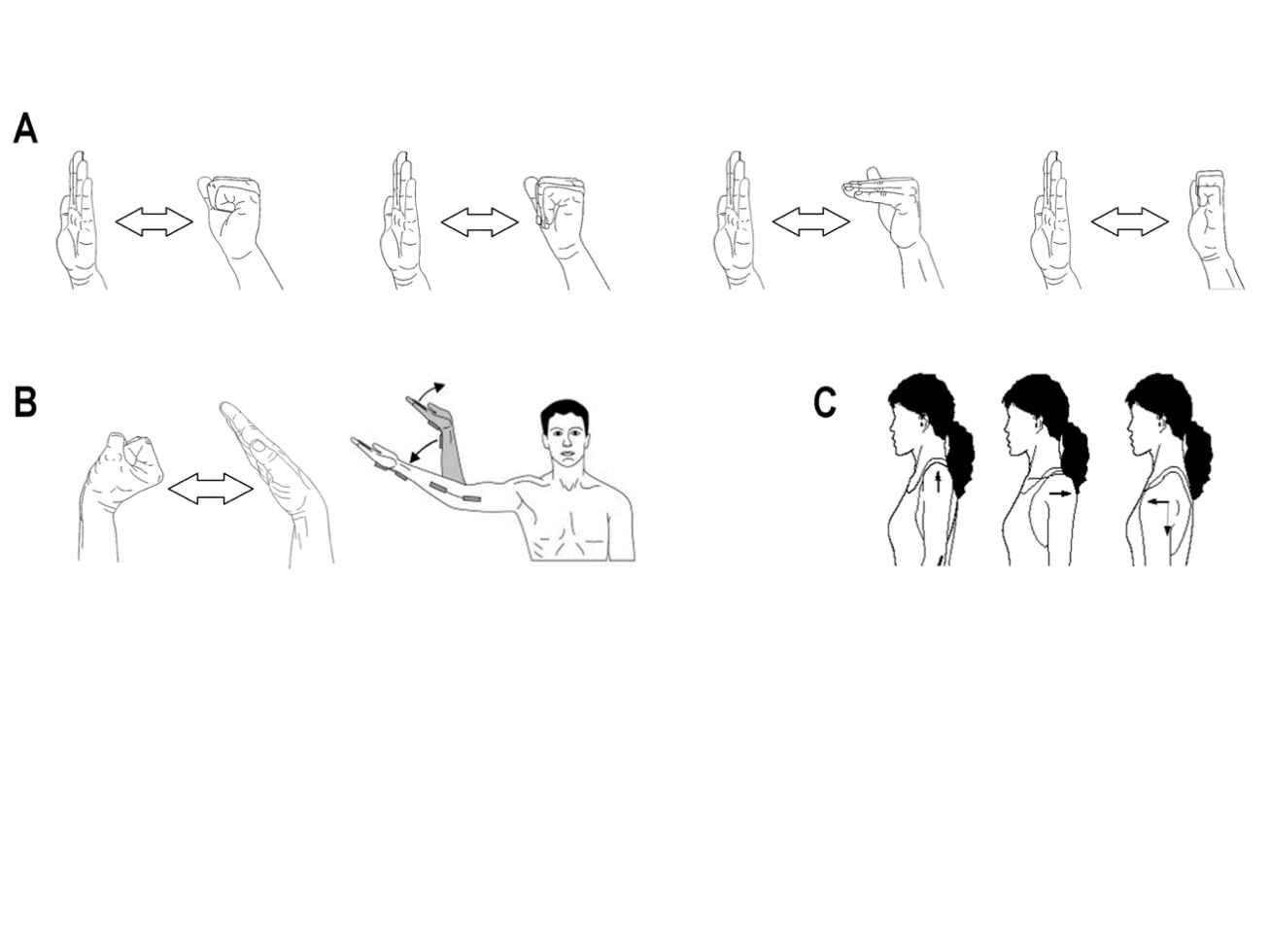

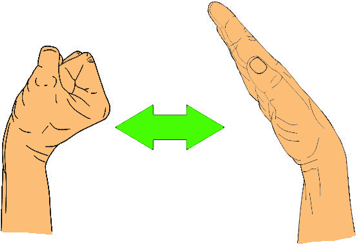


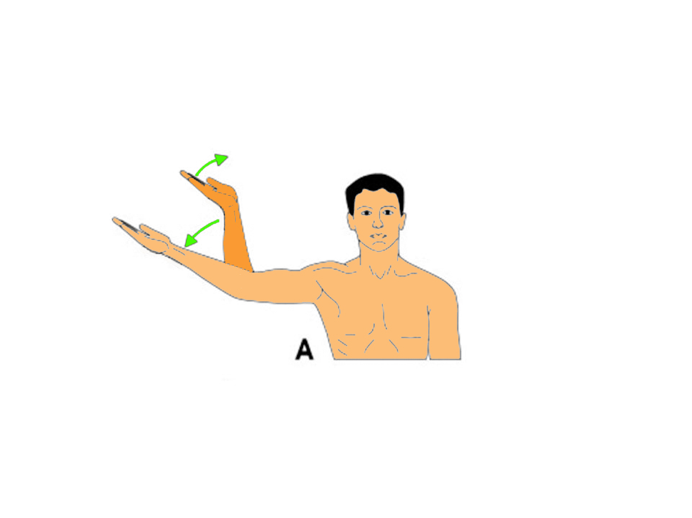

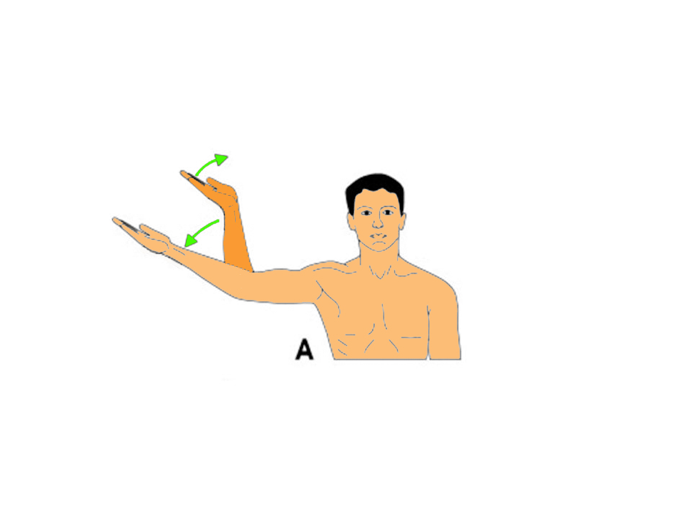

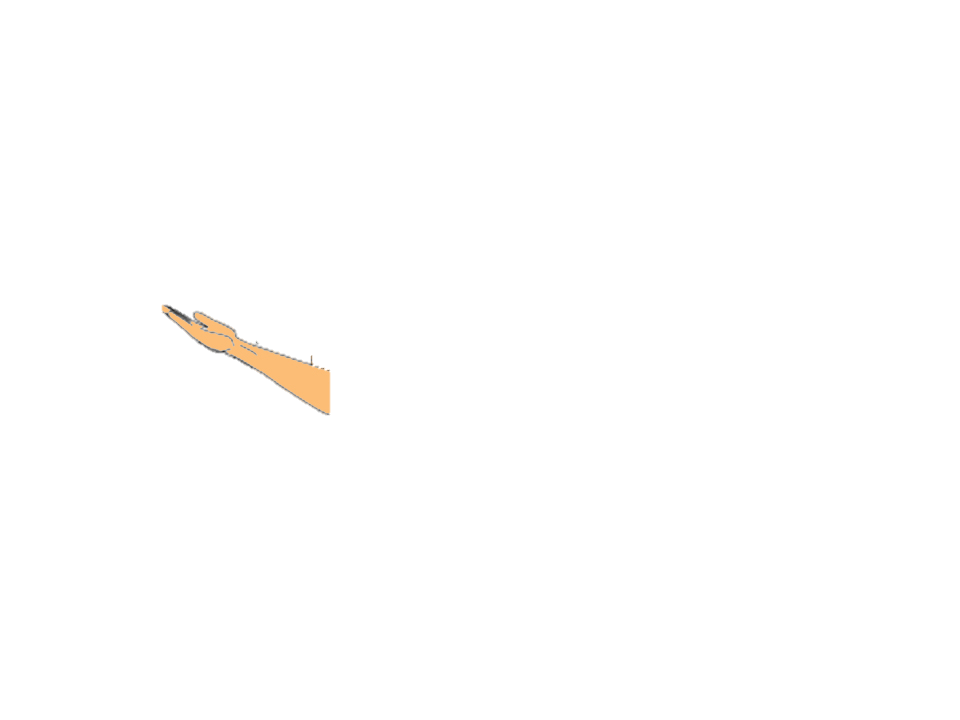


**EXERCISE DIRECTIONS: SUMMARY**

**How many?**

10 repetitions of each exercise per session.

**How often?**

6 sessions/day. Distribute the sessions evenly over the morning, afternoon and evening, leaving 1 hour between sessions.

**How fast?**

1 repetition/second (opening and closing hand is 1 rep).

**How gentle?**

It is all about movement, not strength. Do the exercises gently, but reasonably quickly.

**If you feel an increase in your pain or symptoms you should stop that exercise and contact us!**

**3. Shoulder circles**

**2. Nerve Tensioner: ‘drop the ball’**

**1. Nerve Glide: ‘waiter’**

**8. Tendon Glide: ‘monkey grip’**

**7. Tendon Glide: ‘table top’**

**6. Tendon Glide: fist with straight fingers**

**5. Tendon Glide: fist**


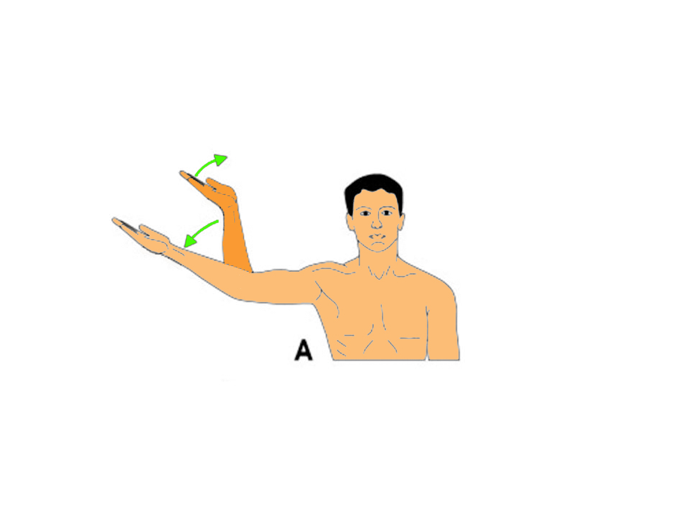

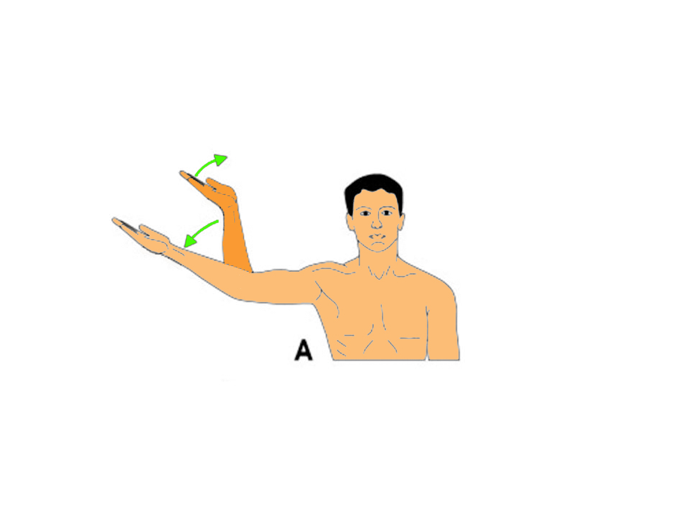

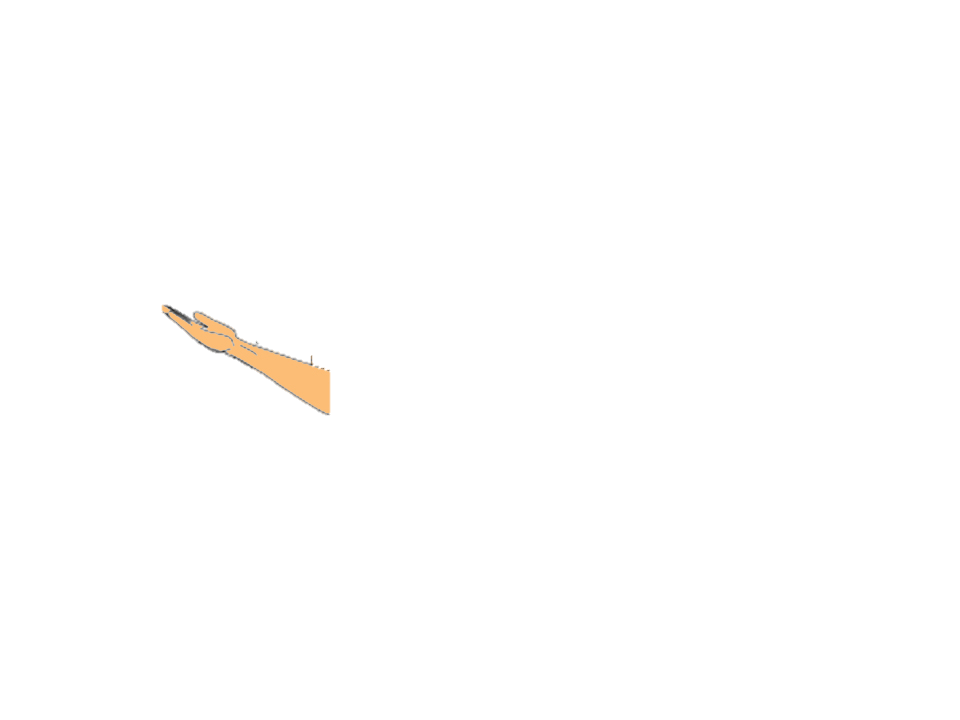

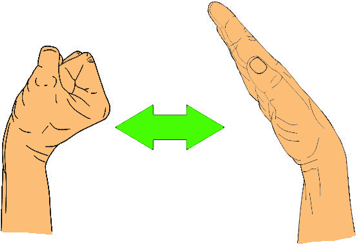


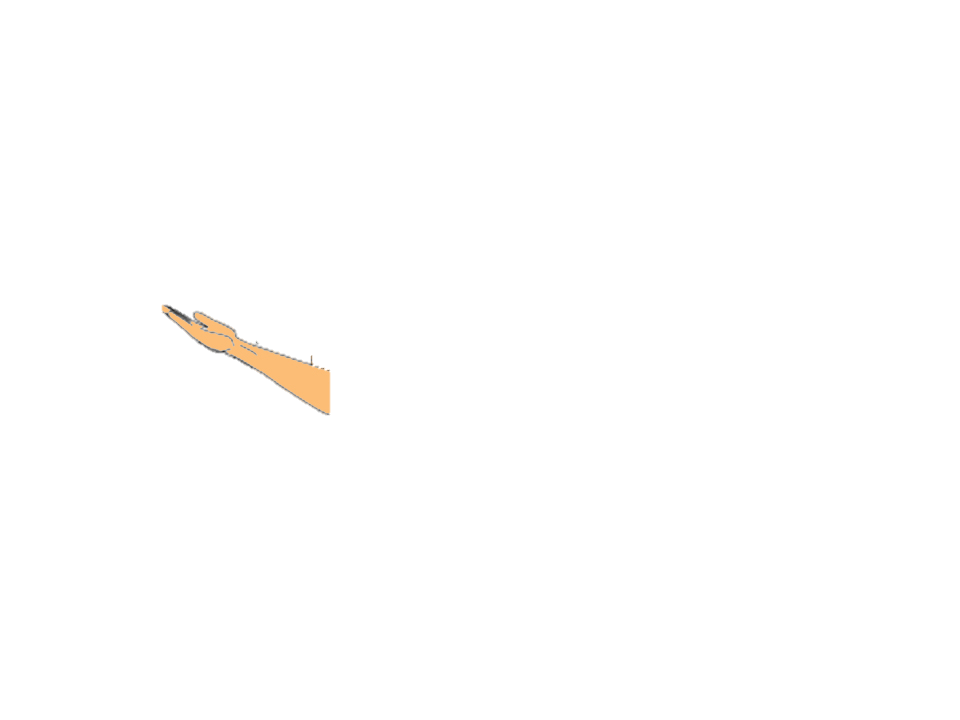

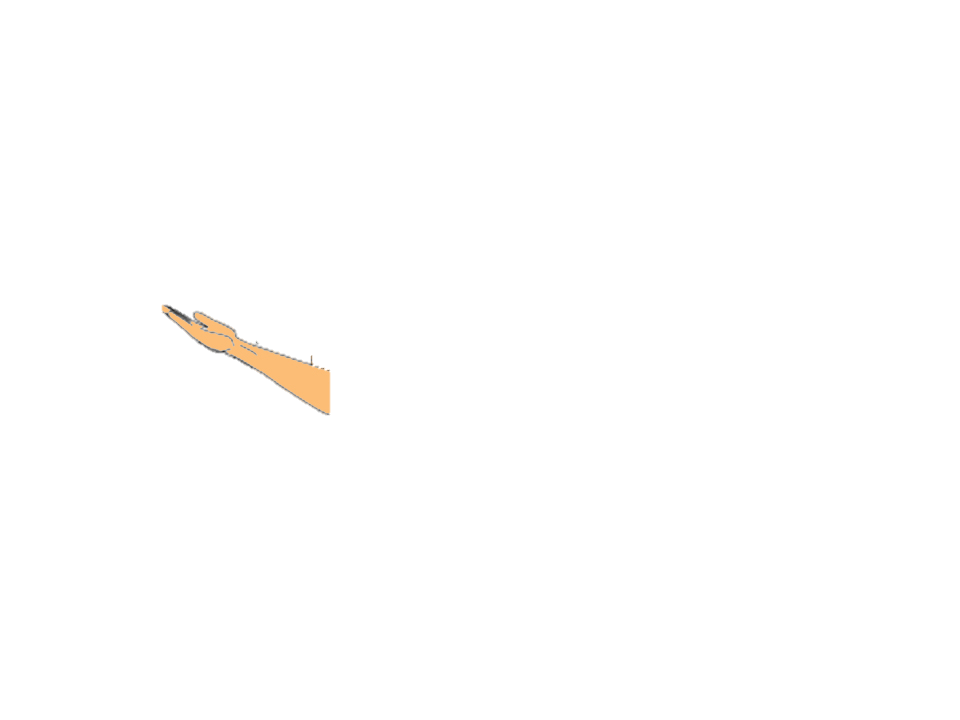


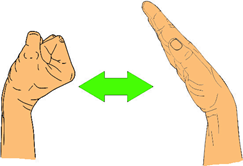


**4. Nerve Glide: ‘throw and catch a ball’**

**WEEK 5-6**

**Neurodynamic exercises summary: take me everywhere!**
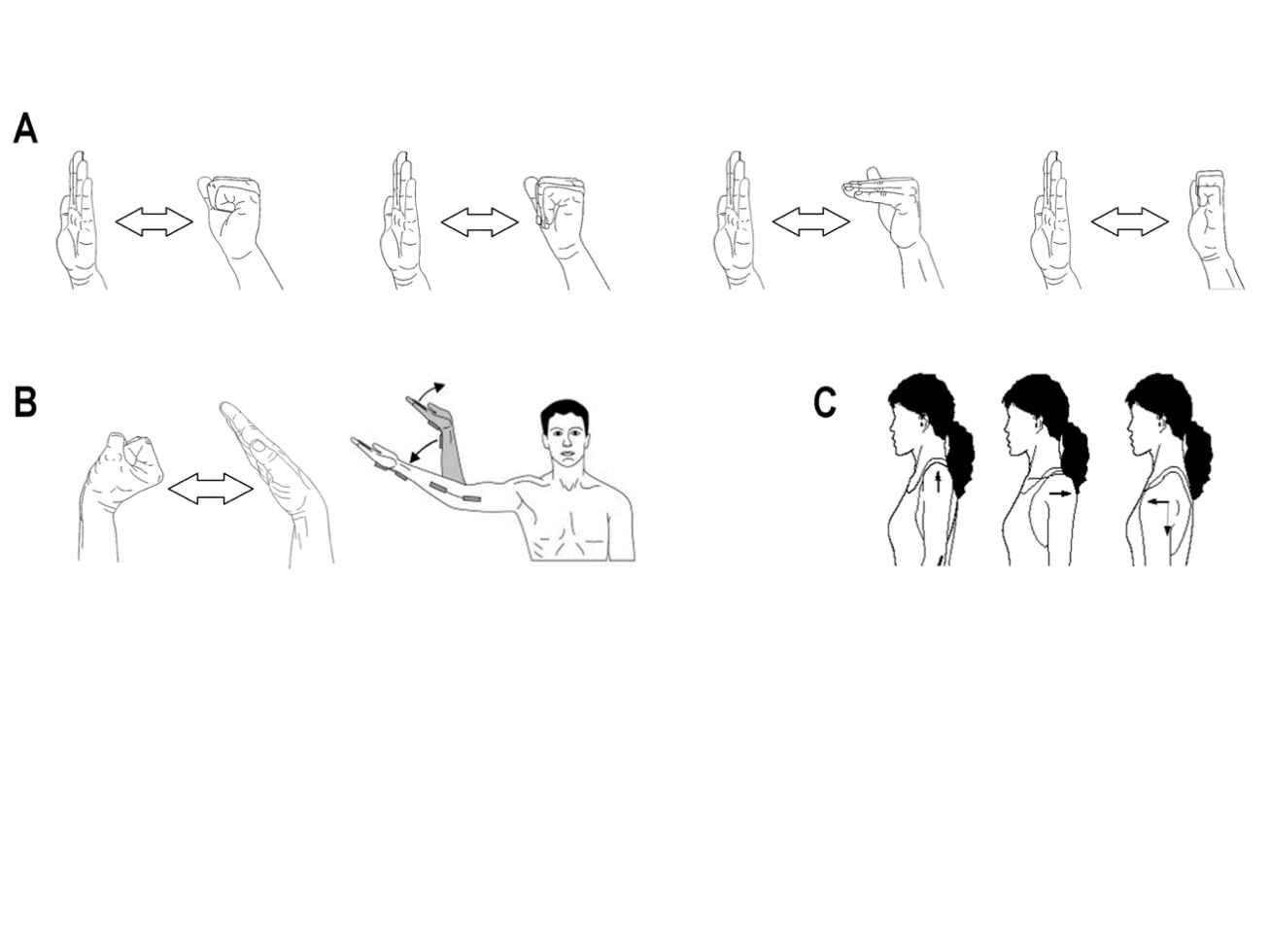

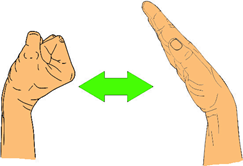

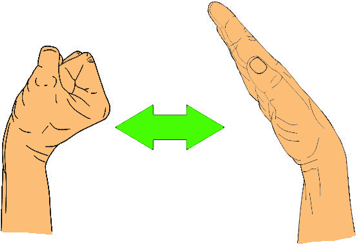


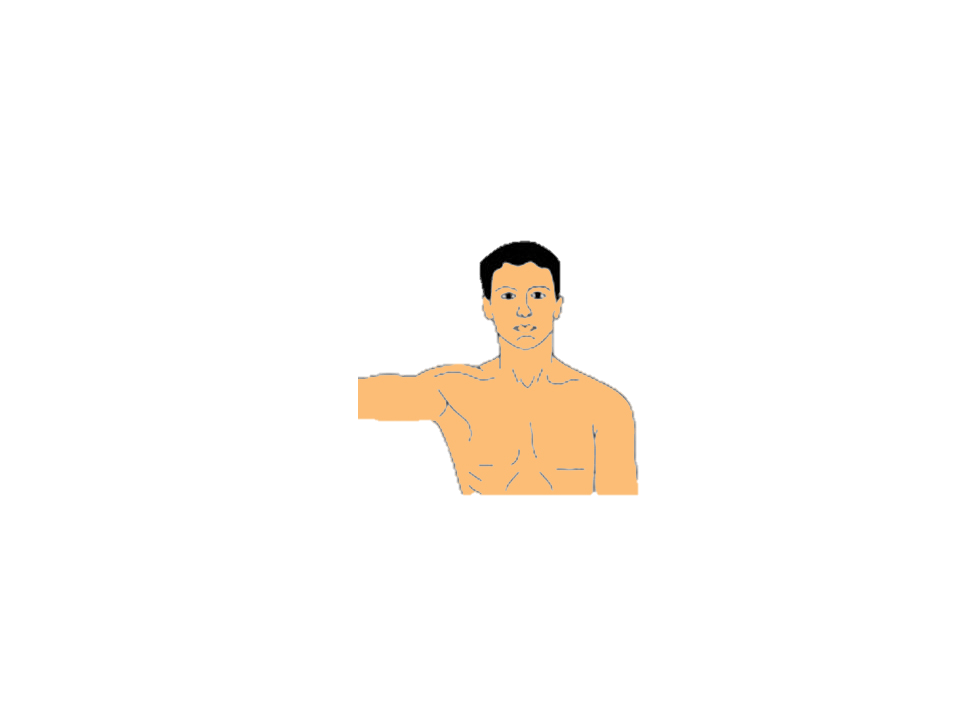

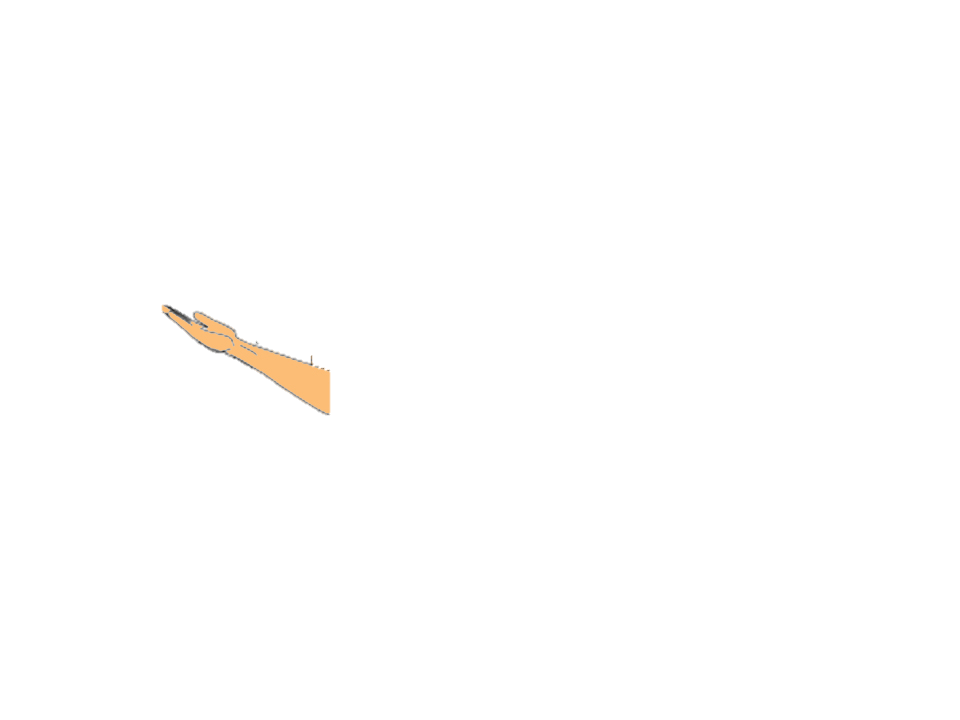

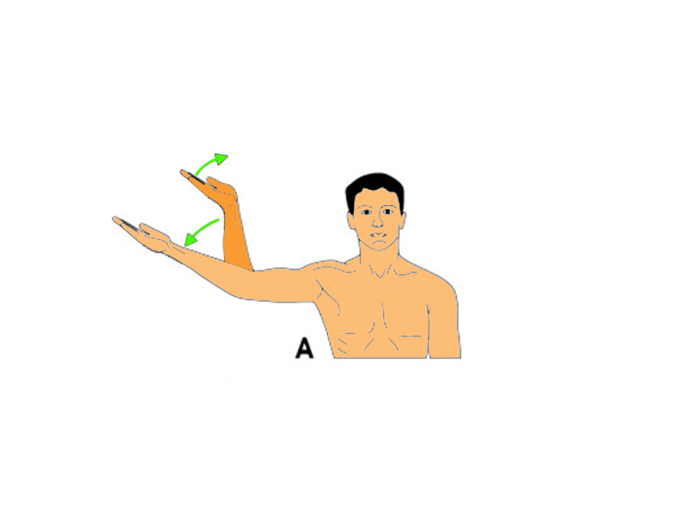

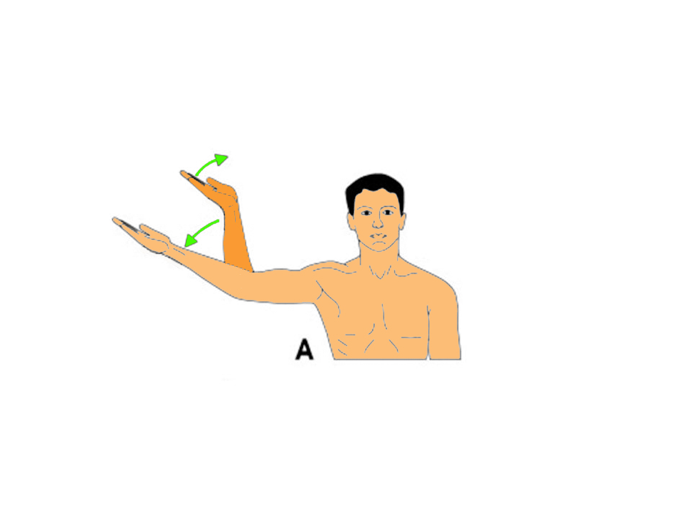

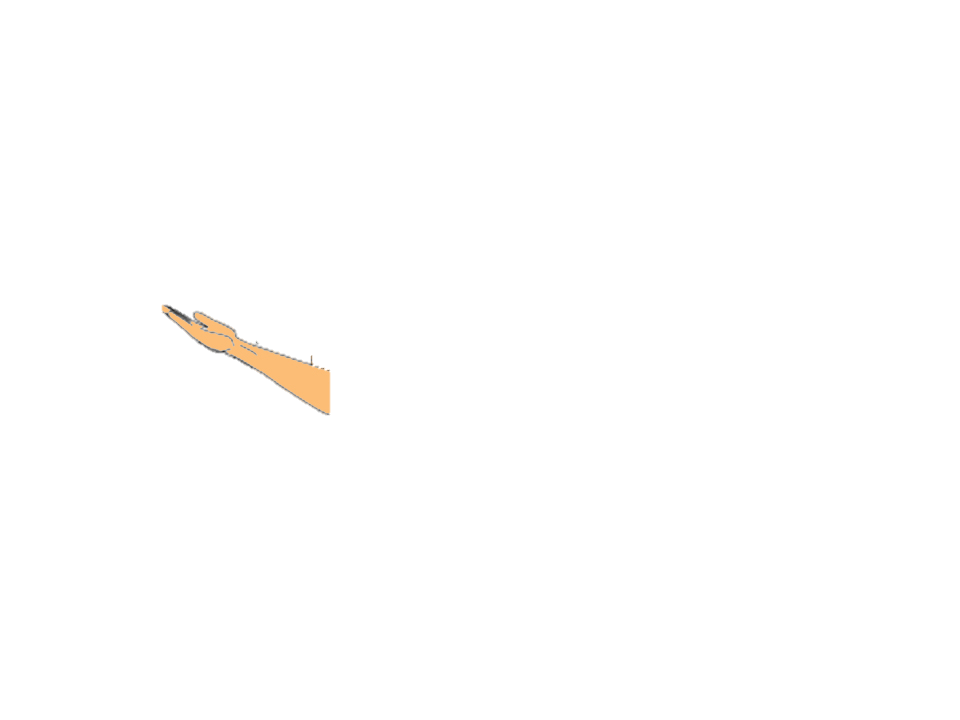

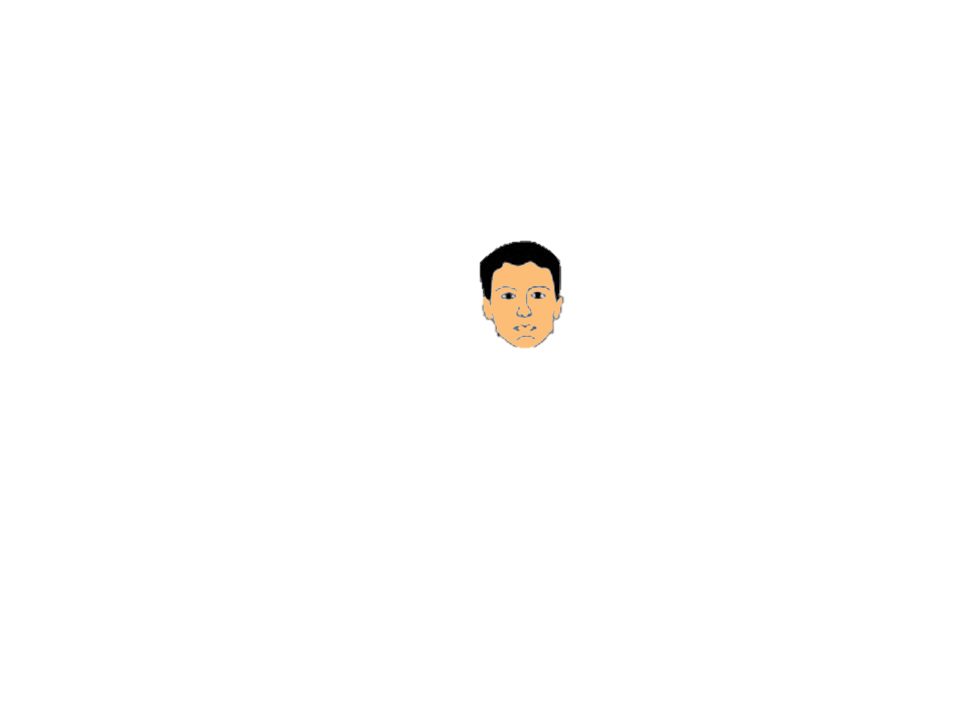


**EXERCISE DIRECTIONS: SUMMARY**

**How many?**

10 repetitions of each exercise per session.

**How often?**

6 sessions/day. Distribute the sessions evenly over the morning, afternoon and evening, leaving 1 hour between sessions.

**How fast?**

1 repetition/second (opening and closing hand is 1 rep).

**How gentle?**

It is all about movement, not strength. Do the exercises gently, but reasonably quickly.

**If you feel an increase in your pain or symptoms you should stop that exercise and contact us!**

**4. Nerve Glide: ‘throw and catch a ball’**

**3. Shoulder circles**

**2. Nerve Tensioner: ‘drop the ball’**

**1. Nerve Glide: ‘waiter’**

**8. Tendon Glide: ‘monkey grip’**

**7. Tendon Glide: ‘table top’**

**6. Tendon Glide: fist with straight fingers**

**5. Tendon Glide: fist**


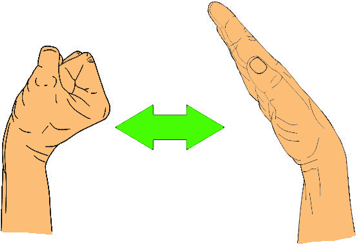


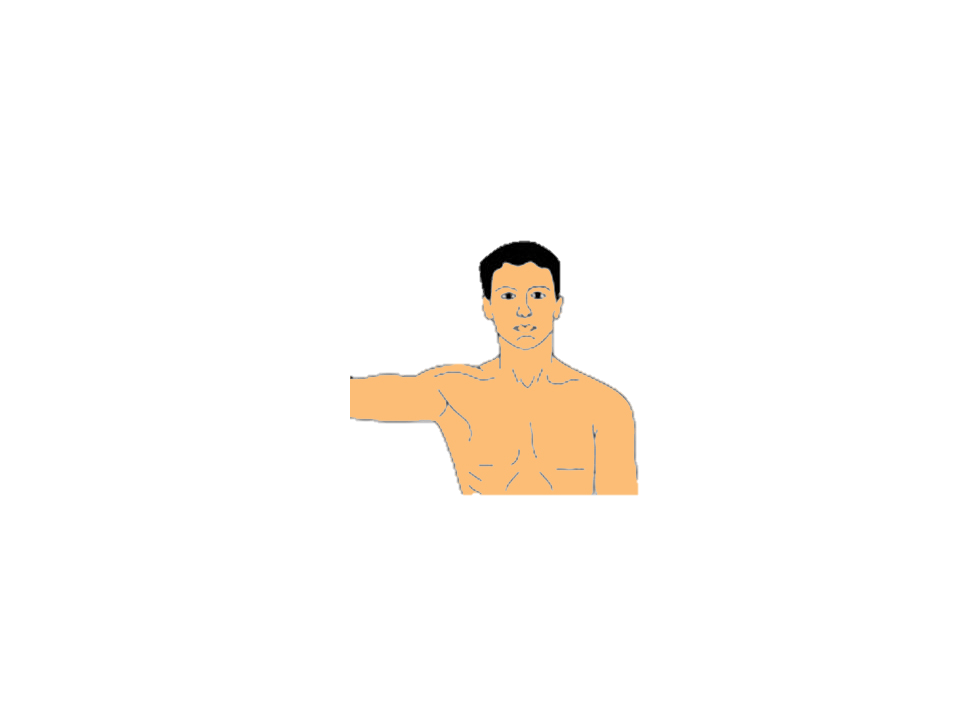

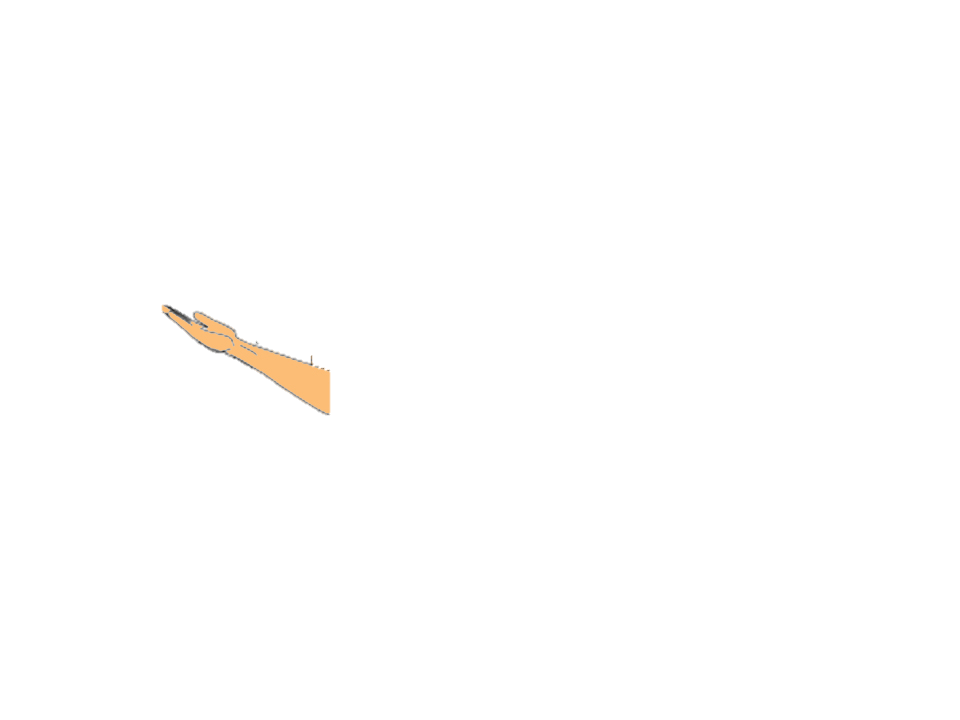

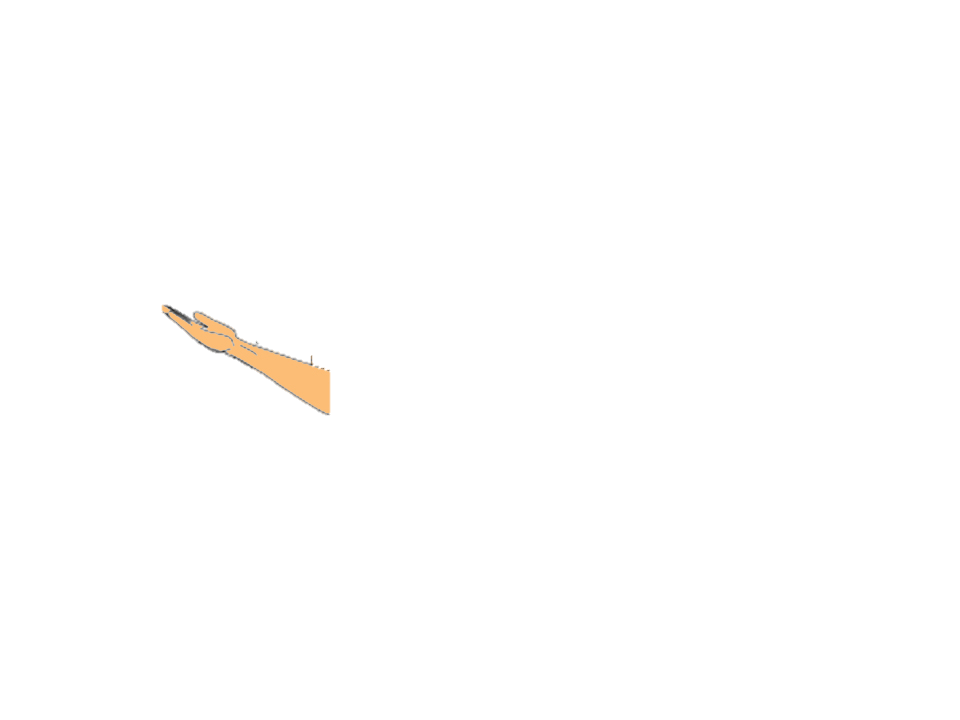

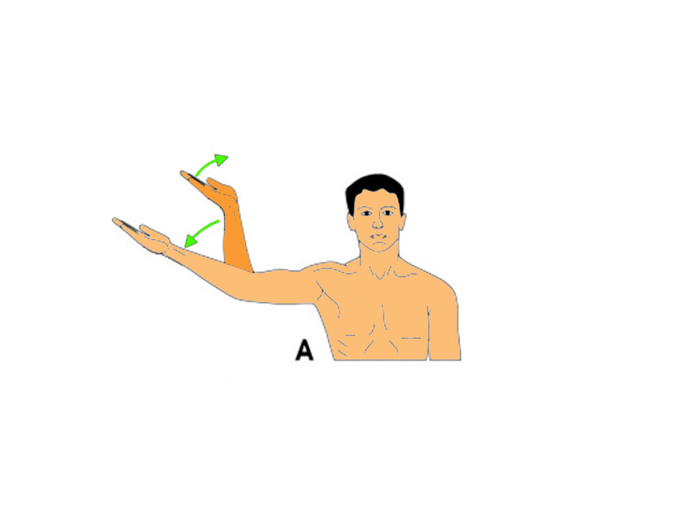

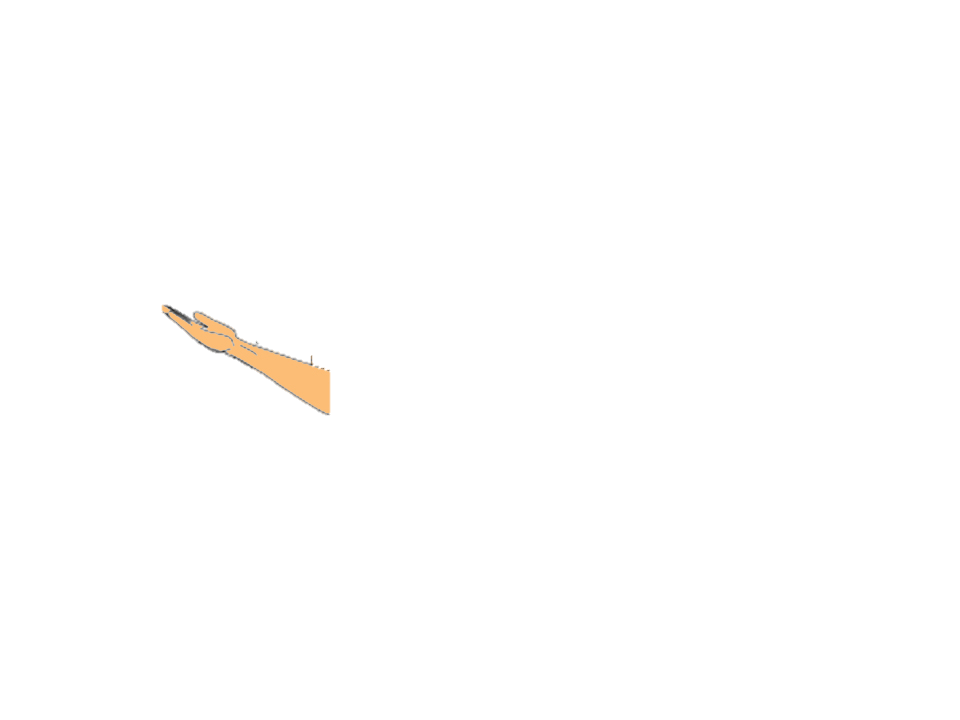

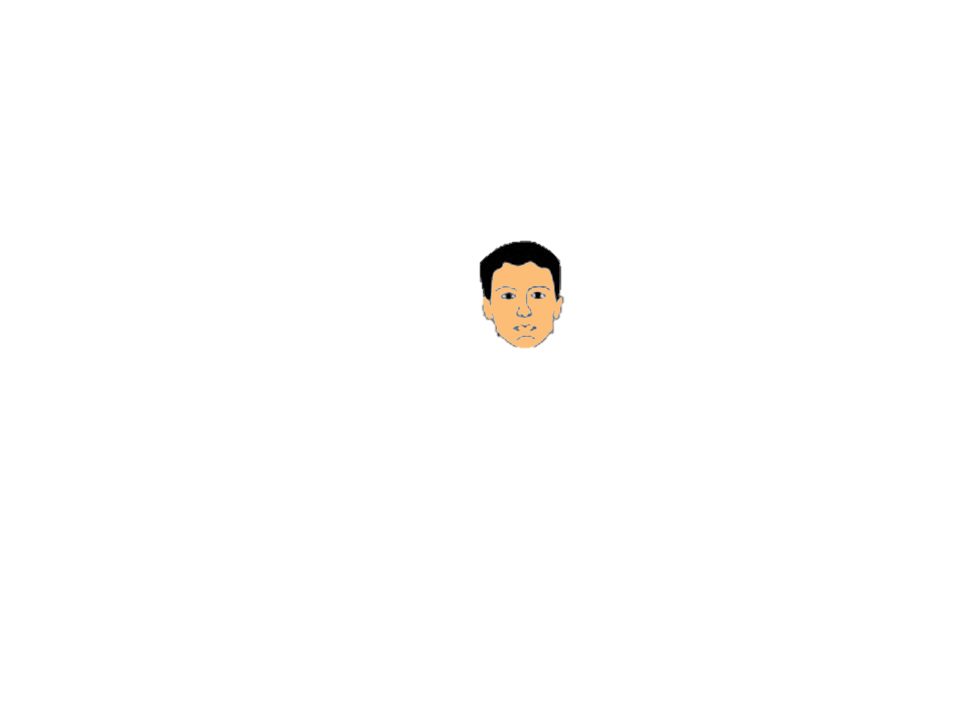


**B. Leaflet for participants allocated to the injection group**

**What is it?**

Carpal Tunnel Syndrome (CTS) is a condition caused by the median nerve being compressed as it passes though the carpal tunnel in the wrist. The carpal tunnel is narrow and is formed by the wrist bones and a thick ligament, as shown in the picture below. CTS affects more women than men and is most common in those aged between 40 and 65.


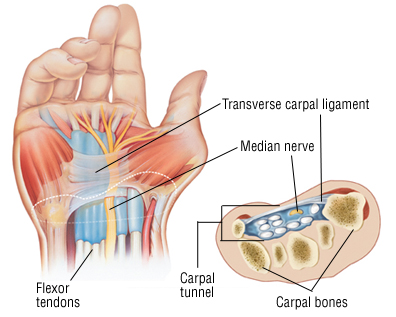


http://www.drugs.com/health-guide/images/205264.jpg

CTS can cause a variety of symptoms, including pain, tingling, numbness, swelling, weakness or clumsiness of the thumb, index, middle and ring fingers. Symptoms are often worse overnight, but may also occur throughout the day. Any condition that reduces the amount of space within the carpal tunnel can cause CTS. Examples of these can include (but are not limited to): inflammation, wrist arthritis, wrist fractures, fluid retention, diabetes or rheumatoid arthritis.

**Steroid injection**

You have been allocated to the steroid injection group. Steroid injections are a routine treatment for people with carpal tunnel syndrome. Corticosteroid is a medicine which can relieve inflammation, swelling, stiffness and pain. Studies have shown that steroid injections can reduce pain and symptoms for patients with carpal tunnel syndrome.

**Is it the same drug that bodybuilder take?**

No. These steroid injections are completely different.

**How is the injection done?**

You will receive a single steroid injection by a trained clinician. The skin will be cleaned with an antiseptic. A needle will be inserted into the carpal tunnel and the steroid solution will be applied through the needle.

**What are possible side effects?**

Side effects are very uncommon; however, you may experience the following:

- A flare-up of your pain within the first 24-48 hours of the injection. This is nothing to worry about and usually settles on its own. Take painkillers (e.g, paracetamol) during this time.
- Flushing of the face, but this generally resolves within 48 hours
- A change in colour or dimpling of the skin around the injection site
- Infection following an injection is rare. However, if the injection site becomes hot, swollen and more painful you should see your GP urgently and contact the study team 01865234821, especially if you feel unwell or feverish.

**How long will it take to work?**

The steroid injection may take up to a week to become effective. There may be some short-lasting discomfort or pain caused by the injection, but this is usually relieved within a few hours.

**What should I do after the injection?**

After the injection, please try and rest your hand for the rest of the day and then resume your normal activities within limits of comfort the following morning.

| **IMPORTANT:**   - Please do not start any other treatments or new exercises for carpal tunnel syndrome during the 6 week intervention period. - If you have any concerns, please contact the study team on <email> or <phone> |
| --- |

**C. Leaflet for participants allocated to the advice group**

**What is it?**

Carpal Tunnel Syndrome (CTS) is a condition caused by the median nerve being compressed as it passes though the carpal tunnel in the wrist. The carpal tunnel is narrow and is formed by the wrist bones and a thick ligament, as shown in the picture below. CTS affects more women than men and is most common in those aged between 40 and 65.


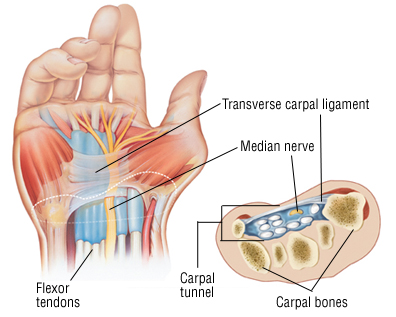


http://www.drugs.com/health-guide/images/205264.jpg

CTS can cause a variety of symptoms, including pain, tingling, numbness, swelling, weakness or clumsiness of the thumb, index, middle and ring fingers. Symptoms are often worse overnight but may also occur throughout the day. Any condition that reduces the amount of space within the carpal tunnel can cause CTS. Examples of these can include (but are not limited to): inflammation, wrist arthritis, wrist fractures, fluid retention, diabetes or rheumatoid arthritis.

**Advice**

You have been allocated to the advice group. In this group, we will ask you to continue with your normal activities as usual. We are interested to find out how our normal daily activities influence the function and structure of your nerve over a short, six-week period. Please do not start any new treatments for carpal tunnel syndrome including medication or physiotherapy during this time.

At the end of the 6-weeks study period, we will offer you one of the other two interventions for free if you wish so.

| **IMPORTANT:**   - Please do not start any other treatments or new exercises during the 6 week intervention period. - If you have any concerns, please contact the study team on <email> or <phone> |
| --- |
